# Na,K-ATPase α1 and β-subunits show distinct localizations in the nervous tissue of the large milkweed bug

**DOI:** 10.1007/s00441-022-03580-6

**Published:** 2022-03-25

**Authors:** Marlena Herbertz, Sönke Harder, Hartmut Schlüter, Christian Lohr, Susanne Dobler

**Affiliations:** 1 Institute of Cell and Systems Biology of Animals, Molecular Evolutionary Biology, Universität Hamburg, 20146 Hamburg, Germany; 2 Institute of Clinical Chemistry and Laboratory Medicine, University Medical Center Hamburg-Eppendorf, 20246 Hamburg, Germany; 3 Institute of Cell and Systems Biology of Animals, Neurophysiology, Universität Hamburg, 20146 Hamburg, Germany

**Keywords:** Insect Na + /K + ‐ATPase, Alpha/beta complexes, Thoracic ganglia, Blood–brain barrier, Protein distribution

## Abstract

The Na,K-ATPase (NKA) is an essential ion transporter and signaling molecule in all animal tissues and believed to consist at least one α and one ß-subunit to form a functional enzyme. In the large milkweed bug, *Oncopeltus fasciatus*, adaptation to dietary cardiac glycosides (CGs), which can fatally block the NKA, has resulted in gene duplications leading to four α1-subunits. These differ in sensitivity to CGs, but resistance trades off against ion pumping activity, thus influencing the α1-subunits’ suitability for specific tissues. Besides, *O. fasciatus* possesses four different ß-subunits that can alter the NKA's kinetics and should play an essential role in the formation of cellular junctions.

Proteomic analyses revealed the distribution and composition of α1/ß-complexes in the nervous tissue of *O. fasciatus.* The highly CG-resistant, but less active α1B and the highly active, but less resistant α1C predominated in the nervous tissue and co-occurred with ß2 and ß3, partly forming larger complexes than just heterodimers. Immunohistochemical analyses provided a fine scale resolution of the subunits’ distribution in different morphological structures of the nervous tissue. This may suggest that α1 as well as ß-subunits occur in isolation without the other subunit, which contradicts the present understanding that the two types of subunits have to associate to form functional complexes. An isolated occurrence was especially prominent for ß3 and βx, the enigmatic fourth and N-terminally largely truncated ß-subunit. We hypothesize that dimerization of these ß-subunits plays a role in cell–cell contacts.

**Supplementary Information:**

The online version contains supplementary material available at 10.1007/s00441-022-03580-6.

## Introduction

The repeated evolution of target-site insensitivity of Na,K-ATPase (NKA) to cardiac glycosides provides an exemplary paradigm for studying the biochemical mechanisms associated with molecular resistance. The NKA is a vital transmembrane enzyme that is ubiquitously expressed in all animal cells. The enzyme’s roles in physiological homeostasis are vast, including maintenance of membrane potentials, septate junction formation, control of cell volume, pH homeostasis, and cell signaling (Horisberger 2004; Pierre and Xie 2006; Paul et al. 2007; Nie et al. 2020). Cardiac glycosides (CG) are natural inhibitors of the NKA and can thus have adverse health effects on whole-organismal physiology (Agrawal et al. 2012; Mohammadi et al. 2017; Züst et al. 2019). These compounds are synthesized in genera of numerous plant families (e.g., Apocynaceae, Plantaginaceae, Ranunculaceae, Iridaceae) and in some animals as a defense strategy (Pasteels and Daloze 1977; Hollman 1985; Krenn and Kopp 1998; Steyn and Van Heerden 1998). Different animals that are exposed to CGs through their diet have independently evolved various amino acid substitutions in the CG binding site of the enzyme (Dobler et al. 2012; Zhen et al. 2012; Ujvari et al. 2015; Mohammadi et al. 2016). In several animals, the evolution of CG resistance is accompanied with gene duplications (Zhen et al. 2012; Petschenka et al. 2017; Dobler et al. 2019; Yang et al. 2019), which can help counterbalance reduced enzyme activity caused by resistance-conferring amino acid substitutions (Dalla and Dobler 2016; Dalla et al. 2017; Mohammadi et al. 2021).

Although our understanding of the molecular mechanisms of this adaptation has significantly advanced in recent years, we lack an understanding of the function of varying quaternary protein structures that can modulate target-site insensitivity and adapt it to tissue specific needs. This question can be addressed by studying an animal with multiple NKA subunit gene duplications resulting in protein isoforms with the ability to build multiple functional enzymes. We therefore decided on studying the large milkweed bug *Oncopeltus fasciatus*.

The large milkweed bug displays intricate adaptations to tolerate the CGs of its *Asclepias* (Apocynaceae) host plants and even sequesters the dietary toxins in its own anti-predator defenses (Scudder et al. 1986; Bramer et al. 2015). The NKA of *O. fasciatus* nervous tissue is highly resistant to CGs (Moore and Scudder 1986; Bramer et al. 2015) due to various amino acid substitutions in the CG target site (Dobler et al. 2012; Zhen et al. 2012). To balance the requirements of CG resistance and effective ion transport, the NKA α1 of *O*. *fasciatus* has undergone several rounds of gene duplication (Zhen et al. 2012) and expresses three α1 paralogs (A, B, C) that may associate with up to four ß-subunits (ß1, ß2, ß3, ßx) to form a functional enzyme (Herbertz et al. 2021). A fourth α1 paralog (D) has a very low expression level (Yang et al. 2019) and has been omitted from the present investigations.

Previous functional tests have shown that the nine possible α1/β-constructs of *O. fasciatus* exhibit different kinetics and CG resistance (Dalla et al. 2017; Herbertz et al. 2021). For example, increased resistance comes at a cost—the strongly resistant subunits αA and αB have reduced ion pumping activity while the highly active α1C subunit exhibits lower CG-resistance (Herbertz et al. 2021; Dalla et al. 2017). Quantitative real-time PCR studies suggest that these different copies are expressed in tissue-specific patterns in *O. fasciatus* (Zhen et al. 2012; Lohr et al. 2017)*.*

So far, it remains unknown whether mRNA expression levels mirror abundances at the protein level and which α1/β subunit combinations are present in the tissues of *O. fasciatus.* The nervous tissue is a good candidate for further investigations, due to the high NKA abundance and high enzyme activity (Lohr et al. 2017; Dalla et al. 2017). However, it is still not clear to which extent CGs are able to enter the nervous tissue, making the existence of a resistant NKA necessary. We address the open questions by characterizing the abundance and distribution of α1/β complexes in the nervous tissue of *O. fasciatus* where the NKA is most important. Specifically, we determined (1) which α1- and β-subunits are present in the nervous tissue, (2) which α1/β-combinations occur, and (3) we tested whether the αCβ3 combination, which was previously inferred to be the most suitable complex for the nervous tissue (Herbertz et al. 2021), actually predominates in this organ. Since even less is known about the four β-subunits, we especially wanted to characterize their occurrence in the nervous tissue and determine whether there are differences in the distribution of the β-subunits among the different structures and cells.

To achieve these aims, we first used 2D-gel electrophoresis (blue native (BN) PAGE/tris tricine PAGE) and immunoprecipitation (IP) with magnetic beads crosslinked to β-subunit-specific antibodies to reveal the occurrence of different α1/β subunits in different cells. We then followed this with LC–MS/MS analyses to determine which α1-subunits dimerize with which β-subunits to form α1/β complexes. Finally, we used immunohistochemistry (IHC) to visualize the structure and cell-specific abundances of different β-subunits.

## Material and methods

### Rearing of *Oncopeltus fasciatus*

The first stock of *O. fasciatus* originated from Ithaca, New York where large milkweed bugs were collected in 2010 (Lohr et al. 2017). To increase genetic diversity and avoid inbreeding depression, new large milkweed bugs were added in 2014 from Urbana-Champaign, Illinois, and in 2019 from the Aquazoo Düsseldorf, Germany. *O. fasciatus* was reared on sunflower seeds in a climate chamber set to 25 °C, 50% humidity and a 14/10 h day/night rhythm throughout the year. All the experiments were conducted with adult female milkweed bugs to exclude sexual genetic differences.

### Tissue dissection

The large milkweed bugs were killed by freezing at − 80 °C. Brains and thoracic ganglia were dissected with two forceps under ice cold highly pure water containing protease inhibitor cocktail (Roche, Basel, Switzerland). Depending on the follow-up method, tissues from 15 to 30 individuals were pooled and stored at − 80 °C for further procedures.

### 2D-gel electrophoresis (BN/TT)

#### Protein extraction and isolation via differential centrifugation

A pool of 15 frozen nervous tissues was homogenized in 300 µl of ice-cold homogenization buffer (250 mM sucrose, 20 mM imidazole/HCl, pH 7) containing protease inhibitor cocktail with an all-glass grinder (Wheaton). After 15 min of incubation on ice, the homogenate was centrifuged twice at 9′000 × g at 4 °C for 15 min. The supernatant was transferred to ultracentrifugation tubes, the volume adjusted to 1 ml with homogenization buffer and centrifuged at 100,000 × g at 4 °C for 1 h 10 min. The pellet was solubilized in 150 µl solubilization buffer (50 mM sodium chloride, 50 mM imidazole/HCl (pH 7), 2 mM 6-aminohexanoic acid, 1 mM EDTA) containing protease inhibitor cocktail and 1.25% DDM (n-dodecyl β-D-maltoside). The protein concentration was determined using a NanoDrop spectrophotometer at 280 nm (Thermo Fisher Scientific, Massachusetts, USA).

#### Blue-native PAGE

The blue-native (BN) PAGE cathode and anode buffer were prepared according to the protocol of Wittig et al. (2006). The gel was cast with slight modifications: Instead of using a 48% acrylamide-bisacrylamide mixture with a 32:1 ratio, we used ROTIPHORESE®Gel 40 (37.5:1) (Carl Roth, Karlsruhe, Germany) to cast a 4% stacking gel and a 4–15% gradient separation gel. The gradient BN-PAGE gel was hand-poured as described in Miller et al. (2016) using a Mini-PROTEAN Tetra cell system (Bio-Rad Laboratories, California, USA). Two samples at 35 µl (20 µg) of protein extract (A) were mixed with one droplet of glycerol and 0.5 µl 5% Coomassie G-250 and separated for 30 min at 100 V with a cathode buffer containing 0.02% of Coomassie G-250 (Serva, Heidelberg, Germany) at 4–7 °C. After the samples ran into the gel 3/4 of the dark blue cathode buffer was replaced with clear cathode buffer and the separation continued at 100 V for another 90 to 120 min. All protein complexes remained intact and appeared as a faint smear. The two lanes were excised immediately after the run and equilibrated in 2 × Laemmli buffer (125-mM Tris/HCL (pH6.8), 20% glycerol, 4% SDS, 0.02% bromophenol blue, add 2.3% fresh DTT) by lightly shaking at 37 °C for 30 min.

#### Tris-Tricine PAGE

For the following Tris-Tricine PAGE, buffers and gels were prepared according to Schägger (2006) with slight modifications. The gels were again poured using a Mini-PROTEAN Tetra cell system with 1.5 mm spacer glass plates, instead of the specified acrylamide/bisacrylamide mixture we used ROTIPHORESE®Gel 40 to prepare a 4% stacking and a 10% separation gel. The separation gel was overlaid with water. After the polymerization was completed, the previously equilibrated gel lane was carefully pushed between the glass plates until no space was left between the separation gel and the gel lane. The residual water was drained off and the gaps were filled up with 4% stacking gel leaving out a cavity for the protein ladder. The electrophoresis was performed at room temperature and started at an initial voltage of 30 V for 1 h. After the sample had entered the separation gel, the voltage was increased to 60 V for 1 h and finally to 100 V until the dye front reached the bottom of the gel (~ 1 h). Two gels were run in parallel. The complex-building NKA α1 and β-subunits were separated from one another by their sizes. After the run, one gel was used for western blotting and the other for silver staining.

#### Western blotting

**Fig. 1:**
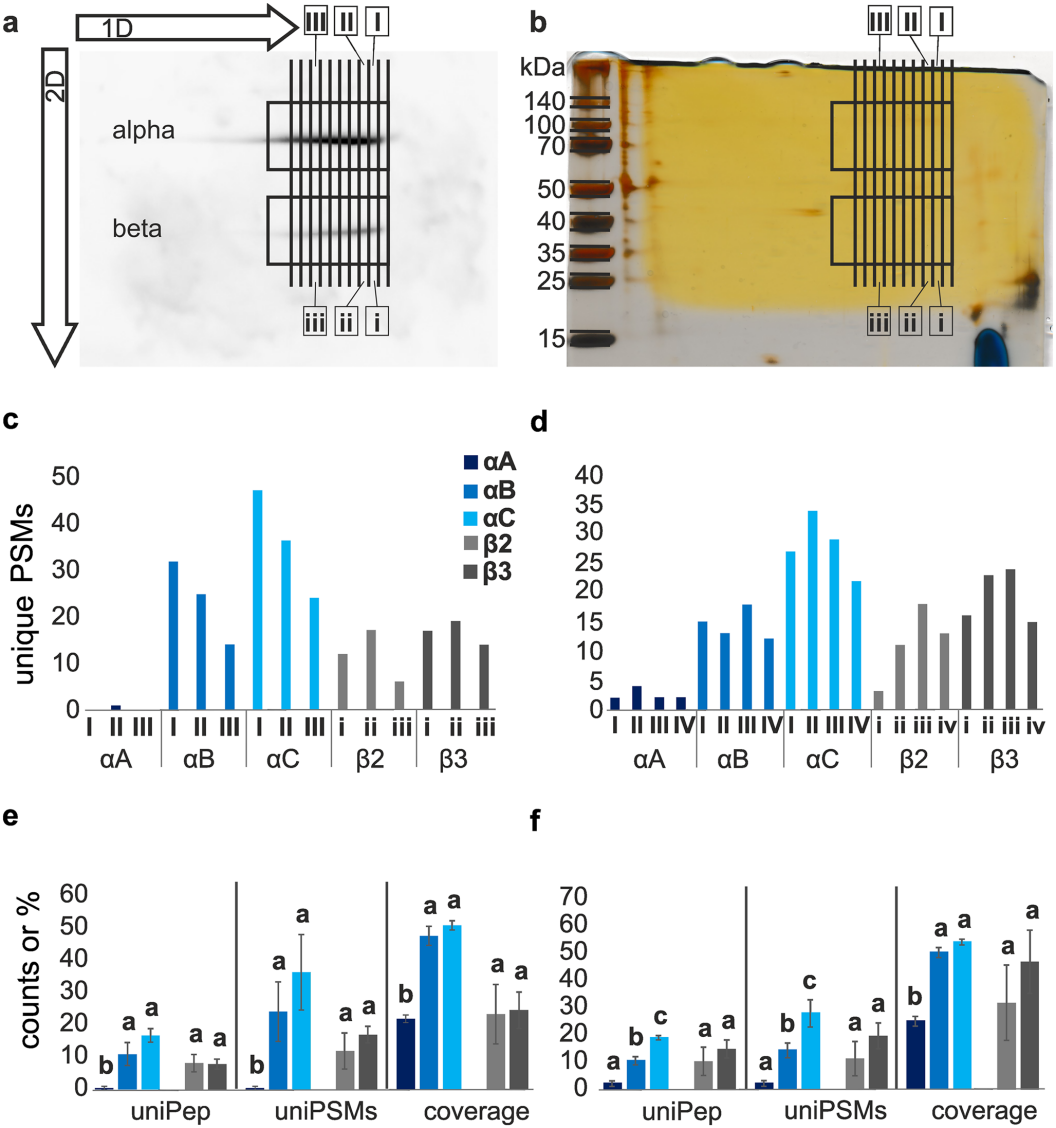
2D-gel electrophoresis followed by LC–MS/MS analyses. **a** Western blot of a 2D-(BN/tris tricine) PAGE. Western blot staining was performed with α5 and Nrv5F7 primary antibody, detected with HRP-conjugated goat-anti-mouse secondary antibody and visualized with ECL. The western blot was used for a better orientation on the corresponding silver stained gel. The large arrows show the running direction of the first (1D) and second dimension (2D). **b** Corresponding silver stained tris tricine gel of nervous tissue proteins after differential centrifugation (gel1). The rectangles mark the α1 (~ 110 kDa) and β-bands (~ 40 kDa) that were cut out of the silver stained gel, the spaces between the lines represent the slices that were cut out. Thin black lines show the samples, which were sent for LC–MS/MS analyses (α1-subunit samples: I, II, III, and corresponding β-subunit samples: i, ii, iii). **c** Analyses of unique peptide-spectrum matches (unique PSMs) originating from different subunits of the NKA (αA, αB, αC, β2, β3) from nervous tissue of *O. fasciatus.* Resulting unique PSMs from LC–MS/MS analyses of samples (*N* = 3 for α and β, respectively) from the 2D-gel 1 (b). **d** Resulting unique PSMs from LC–MS/MS analyses of samples (*N* = 4 for each α and β) from the 2D-gel 2 (Fig. S2). Identified unique PSMs were plotted for every gel sample (α-band: I–IV; β-band i–iv) for each detected Na,K-ATPase subunit. **e** Plot of mean values + / − standard deviation of unique peptides (uniPep), unique PSMs (uniPSMs), both expressed in counts; and total sequence coverage including shared and unique peptides (coverage), expressed in percentage (%), identified in different 2D-gel electrophoresis samples of nervous tissue pool gel 1 (*N* = 3) **f** and gel 2 (*N* = 4). Different letters indicate significant differences across one subunit (α1 or β)

Western blotting was performed as described in Dalla et al. (2017) with few modifications. The membrane proteins were blotted on a nitrocellulose membrane overnight at 30 V and kept cool with an additional ice block at 4 °C. After blocking for 1 h, the membrane was incubated with the primary monoclonal antibodies α5 (binding to NKA α1) and Nrv5F7 (binding to NKA β) in a 1:100 dilution (DSHB Hybridoma Bank, University of Iowa; deposited by D. M. Fambrough (Lebovitz et al. 1989) and P. M. Salvaterra (Sun and Salvaterra 1995), respectively) for 1.5 h at room temperature. For detection of the primary antibodies, the membrane was incubated with the secondary antibody goat anti-mouse conjugated with horseradish peroxidase (Dianova, Hamburg, Germany) and developed with ECL western blotting substrate following the manufacturer’s protocol (Promega, Wisconsin, USA) or 4-chloro-1-naphtol as described in Dalla et al. (2017). With the western blot, the dissociation of NKA α1 and β-subunits was confirmed. It additionally functioned as orientation for the preparation of the silver-stained gel. Two bands appeared right on top of each other on both western blots. The horizontal streaks reflect the presence of different protein isoforms of the α1 and β-subunits, which just slightly differ in their molecular weights (Table S1). The α1-subunits (~ 110 kDa) and the β-subunits (~ 40 kDa) showed a strong signal on the western blots (Fig. 1a; Fig. S2).

#### Silver staining

MS-compatible silver staining was performed based on the protocol of Blum et al. (1987) following the modified version by Mortz et al. (2001). Only faint α1 and β-bands appeared in the corresponding silver-stained gels (Fig. 1b), indicating a low amount of the NKA in comparison to proteins that are present with strongly stained bands.

#### Sample preparation for LC–MS/MS analysis

The silver-stained gel was placed on a foil-wrapped graph paper to ensure a perfectly straight orientation. The horizontal α1 and β-lane was punched in a rectangular shape but was not removed from the gel. Thin gel pieces of precisely 1-mm width of the α1 and β-lanes were cut out vertically with a set of nine razor blades fixed with 1-mm spacers in between (Fig. S1). The resulting gel slices contained subunits that were derived from a single complex (Fig. 1a, b; Fig. S2). Each gel slice was transferred into a low bind 0.5 ml reaction tube and three to four samples (α-band: I, II, III, and IV; β-band: i, ii, iii, iv) were subjected to tryptic in-gel digestion.

#### Tryptic in-gel digestion

Following Shevchenko et al. (2007) in-gel digestion was performed. Shrinking and swelling was done with 100% acetonitrile (ACN) and 100 mM NH_4_HCO_3_. The disulfide bonds of proteins were reduced with 10 mM dithiothreitol (dissolved in 100-mM NH_4_HCO_3_). The SH-groups were alkylated with 55 mM iodacetamide, dissolved in 100 mM NH_4_HCO_3_. By covering the gel pieces with a trypsin solution (8 ng/µl sequencing-grade trypsin, dissolved in 50 mM NH_4_HCO_3_) and incubating the mixture at 37 °C overnight, the proteins were hydrolyzed. Tryptic peptides were extracted into the supernatant by adding 2% FA (formic acid) in 80% ACN. The supernatant was transferred into a new vial and evaporated. After dissolving the tryptic peptides in 20 µl 0.1% FA, the samples were analyzed with liquid chromatography coupled to tandem mass spectrometry (LC–MS/MS).

### Immunoprecipitation with β-specific antibodies coupled magnetic beads

#### Crosslinking of antibodies

Thirty micrograms of previously specificity-validated β1, β2, β3, and βx-primary polyclonal antibodies (Davids Biotechnologie, Regensburg, Germany; Herbertz et al. 2021) were individually crosslinked to 5 µl of Protein A Mag Sepharose beads (GE life science, Marlborough, USA) according to the manufacturer’s protocol with slight modifications. The solution exchanges were performed with the help of a magnetic device provided by the manufacturer. The beads were equilibrated in a 500 µl binding buffer (1.2 M KH_2_PO_4_, pH 9). The antibodies were mixed with at least 4 volumes of binding buffer, added immediately after bead equilibration and incubated overnight at 4 °C and slow rotation. The beads were washed with a binding buffer, equilibrated with 500 µl of 0.2 M triethanolamine (pH 8.9) and finally the crosslinking solution (0.2 M triethanolamine, 50 mM DMP (dimethyl pimelimidate dihydrochloride)) was added and incubated for 1 h at room temperature at slow rotation. Afterwards, the reaction was blocked with 0.1 M ethanolamine (pH 8.9) for 15 min at slow rotation. The non-bound antibody was removed by incubating the magnetic beads with elution buffer (0.1 M glycine, pH 2.9) for 2 min, then washed twice with a binding buffer for 5 min and stored until immunoprecipitation in 20% ethanol at 4–7 °C.

#### Evaluation of antibody binding

Successful binding of antibodies to the magnetic beads was validated by dot blots with at least one dilution (1:10 plus eventually higher dilutions). The signal given by the non-bound antibody solution (after overnight incubation with beads) was compared to the initially added antibody solution. No signal was an indicator for complete binding, a light signal indicated good binding but also a slight excess of antibody and a strong signal (almost as strong as the starting solution) indicated insufficient binding. β1, β2, and βx were successfully crosslinked to the magnetic beads whereas for β3 the non-bound fraction was again incubated overnight with the beads to achieve the same amount of binding (Fig. S3).

#### Protein extraction for IP with specific antibodies

Protein was extracted from 25 pooled nervous tissues in 300 µl RIPA lysis buffer (50 mM Tris/HCl pH 8, 150 mM NaCl, 1% Triton-X-100, 0.5% sodium deoxycholate, 0.1% SDS) containing protease inhibitor cocktail similar to the procedure described before for 2D-gel electrophoresis and incubated for 30 min on ice. After centrifugation at 6000 × g at 4 °C for 10 min, the supernatant was split into two subsamples for further proceedings.

#### Binding of target protein

Four times the volume of binding buffer (pH 7) was added to the two nervous tissue extracts. One extract was incubated with magnetic beads coupled to β2, and the other extract was incubated with beads coupled to β3 overnight at 4 °C with slow rotation. The non-bound fraction was removed and collected for incubation with magnetic beads coupled to β1 and βx, following the same procedure as described above. After three washing steps (with binding buffer pH 7), the bound protein was eluted twice with 50 µl of 0.1 M glycine (pH 2.9) with a slight shaking for 10 min. To recover the native state of the proteins 6 µl of 1 M tris salt was pipetted immediately to the eluate. The beads were washed with PBS-T and PBS and stored in 20% ethanol at 4 °C. The recovered protein concentrations were quantified with the Qubit assay kit (Thermo Fisher Scientific, Massachusetts, USA) following the manufacturer’s instructions. The principle of protein quantitation with the Qubit assay kit is based on the detection of target-specific fluorescence, detectable upon binding of the provided dye to the protein. Dot blots were used to test for successful enrichment of the target proteins β1, β2, β3, and βx. After verifying the enrichment, the protein eluates were subjected to LC–MS/MS analyses (Fig. S4).

#### Tryptic in solution digestion

Samples were dissolved in 100 µl SDC buffer (100 mM triethyl ammonium bicarbonate and 1% w/v sodium deoxycholate). With dithiothreitol (10 mM) disulfide bonds were reduced, resulting SH-groups alkylated in the presence of iodoacetamide (55 mM) and, thereafter, proteins digested with trypsin in a ratio of 1:100 (sequencing grade, Promega) at 37 °C overnight. By the addition of 1% v/v formic acid, SDC was precipitated. After centrifugation at 16,000 g, the supernatant was transferred into a new tube and the tryptic peptides were dried in a vacuum centrifuge. Samples were dissolved in 20 µl 0.1% FA prior to LC–MS/MS analysis.

### LC–MS/MS analysis

For protein identification the tryptic peptides were injected into a LC–MS/MS system, consisting of a nano-liquid chromatography system (Dionex UltiMate 3000 RSLCnano, Thermo Scientific, Bremen, Germany) coupled via electrospray-ionization (ESI) to a tribrid mass spectrometer equipped with a quadrupole, an orbitrap and a linear iontrap (Orbitrap Fusion, Thermo Scientific, Bremen, Germany) or with nano liquid chromatography system (nanoACQUITYy, Waters, Manchester, UK) coupled via ESI to a MS comprising a quadrupole and an orbitrap mass analyzer (Orbitrap QExcactive, Thermo Scientific, Bremen, Germany). The samples were injected (5 µl) with a flow rate of 5 µl/min into the LC–MS/MS system. The peptides were enriched on a trapping column (Acclaim PepMap µ-precolumn, C_18_, 300 µm × 5 mm, 5 µm, 100 Ǻ, Thermo Scientific, Bremen, Germany; nanoACQUITY UPLC Symmetry C_18_ trap column, 180 μm × 20 mm, 5 µm, 100 Ǻ; buffer A: 0.1% FA in HPLC-H_2_O; buffer B: 0.1% FA in ACN) with 2% buffer B. Thereafter the trapping column was washed for 5 min for desalting of the peptides, with 2% buffer B (5 μl/min). Peptides were transferred to (200 nl/min) to a longer column for separation (Acclaim PepMap 100, C_18_, 75 μm × 250 mm, 2 µm, 100 Ǻ, Thermo Scientific, Bremen, Germany; nanoAcquity UPLC column, BEH 130 C_18_, Waters; 75 μm × 250 mm, 1.7 µm, 100 Ǻ; 200 nl/min, gradient: 2 − 30% B in 30 min). The ESI spray was achieved by a fused-silica emitter (I.D. 10 μm, New Objective, Woburn, USA) at a capillary voltage of 1650 V. The positive ion mode was chosen for detecting the peptides. The MS/MS analysis was performed in the data dependent acquisition mode (DDA) selecting the top speed modus. For HCD collision, an energy of 28% was chosen, the intensity threshold was 2 × 10^5^ and the isolation width of 1.6 m/z. Every second over a m/z range from 400 to 1500 (resolution of 120,000 FWHM at m/z 200; transient length = 256 ms; maximum injection time = 50 ms; AGC target = 2 × 10^5^) a MS scan was performed. MS/MS spectra were obtained in the ion trap (scan-rate = 66 kDa/s; maximum injection time = 200 ms; AGC target = 1 × 10^4^). LC–MS/MS analysis with the orbitrap QExcactive was performed on MS level over a m/z range from 400 to 1500 (resolution of 70,000 FWHM at m/z 200; transient length = 256 ms; injection time = 100 ms, AGC target = 3 × 10^6^). MS/MS measurements were done using the DDA mode choosing the following parameters: Top5; HCD collision energy of 30%; resolution of 17,000 FWHM at m/z 200; transient length = 64 ms; injection time = 100 ms; AGC target = 3 × 10^6^; underfill ratio of 10%; isolation width of 2 m/z.

### Data preparation and statistical analyses

#### LC–MS/MS data analysis

LC–MS/MS data were processed with the Proteome Discoverer 2.0 software (Thermo Scientific, Bremen, Germany). For identifying the proteins from the MS/MS spectra, the resulting peptides were compared with the protein sequences of all three α1-subunits and the four β-subunits that were predicted from their gene sequences (European Nucleotide Archive, https://www.ebi.ac.uk/ena/data/view/OW028344-OW028350) in combination with a contaminant database (www.uniprot.org).

For the searches, the following parameters were chosen: Precursor mass tolerance: 10 ppm; fragment mass tolerance: 0.2 Da. Two missed cleavages were allowed. Carbamidomethylation on cysteine residues as a fixed modification and oxidation of methionine residues as a variable modification were selected. Peptides with a FDR of 1% using a Percolator were identified. At least two unique peptides per protein were requested as a condition for a reliable identification.

With MaxQuant (Version 1.5.8.3) (PMID 19,029,910), identification of proteins was performed with Andromeda using the predicted *Oncopeltus fasciatus* protein sequences. The MaxQuant parameters were set as follows: the precursor mass tolerance was set to 10 ppm, the fragment mass tolerance was set to 0.5 Da, and two missed cleavages were allowed for peptide identification; an FDR of 1% was given and a maximum of five modifications per peptide were allowed. As a fixed modification, the carbamidomethylation on cysteine residues and as variable modifications, the oxidation of methionine residues and the acetylation of protein N-terminals were set. The LFQ minimum ratio count was set to 1.

Non-target proteins were excluded from the data set and only peptides matching predicted NKA fragments were retained. The following variables resulting from the LC–MS/MS analyses were taken into account: unique peptides (can only be found in the protein sequence of one subunit, unique for only one subunit), shared peptides (found in more than one protein sequence, common in more than one subunit), PSMs (peptide spectrum matches, total number of identified peptides containing also repeatedly identified ones including both, unique and shared peptides), unique PSMs (shared peptides were subtracted resulting in unique peptide spectrum matches), sequence coverage (percentage of protein sequence covered by detected unique and shared peptides). The higher the values of unique peptides and protein sequence coverage the higher the support for reliability of subunit existence and quantity predictions. The number of unique PSMs was used as an indicator for protein quantity (Madsen et al. 2015).

Differences in amounts of unique peptides, unique PSMs, and sequence coverage (unique and shared peptides) between the detected subunits (αA, αB, αC, β2, and β3) from the two 2D-gels were statistically evaluated by performing analyses of variance (ANOVA) using the mean values. In advance, we tested the data for normal distribution (Shapiro Wilk’s test; *p* > 0.05) and variance homogeneity (Levene’s test; *p* > 0.05). The three variables (unique peptides, unique PSMs, and sequence coverage) were analyzed separately by one-way ANOVA followed by Tukey’s HSD post hoc tests. Here, the mentioned variables were set in dependence of the five subunits. All statistical analyses were performed in R Studio (Version 3.6.3).

### Immunohistochemistry with β-specific antibodies

To fix the large milkweed bugs (killed by freezing) the wings and legs were cut off, and the abdomen and thorax were opened laterally and incubated overnight at 4 °C with 4% paraformaldehyde in phosphate-buffered saline (PBS) (pH 7.4). The large milkweed bugs’ bodies were placed in 3% low melt agarose at a temperature of approximately 35 °C. After complete polymerization, the agarose blocks were trimmed taking care that the large milkweed bug inside had a parallel orientation to the edges.

The rectangular shaped block was glued on the vibratome specimen holder (VT1000S, Leica, Wetzlar, Germany). The specimen bath was filled with PBS buffer and covered the agarose block completely. Coronal slices of 300 µm width were produced continuously from head to metathorax. The slices were collected in glass dishes and washed three times in PBS for 5 min. Next, the slices were incubated overnight in a blocking solution (2.5% BSA, 2.5% NGS, 0.25% Triton X-100 in PBS) followed by incubation with primary antibodies α5 and either β1, β2, β3, or βx (5 µg/ml; 10 µg/ml for β1) in a blocking solution (1:5 dilution) for 3 days at 4 °C with slow end-to-end movement. After three washing steps, the secondary antibodies goat anti-mouse Cy3 (1:200 in 1:5 diluted blocking solution; Sigma, St. Louis, Missouri, USA), goat anti-chicken Alexa 405 (1:1000 in 1:5 diluted blocking solution; Sigma, St. Louis, Missouri, USA) and goat anti-rabbit Alexa 488 (1:1000 in 1:5 diluted blocking solution; Invitrogen, Carlsbad, California, USA) were incubated for another 2 days at 4 °C. After washing the slices thoroughly with PBS they were transferred on object slides, embedded with air-hardening Shandon Immu-Mount (Thermo Scientific), covered with coverslips and sealed the next day with nail polish. Immunohistological stainings were imaged using a confocal microscope eC1 (Nikon, Düsseldorf, Germany) equipped with a 40 × lens (CFI Plan Fluor 40 × Oil, NA 1.3, Nikon). The gain values for each fluorescence channel were adjusted to match that of the negative controls. Background was subtracted with the function “subtract background” using the “rolling ball” algorithm in Fiji ImageJ 1.53c (Fiji, RRID:SCR_002285). The algorithm removes an averaged local background from the image to correct for intensity variations without eradicating intensive pixels and without the need for a control image (Sternberg 1983). Each sample set was accompanied by a negative control prepared without primary antibody.

### Results

#### Mass spectrometric approach (LC–MS/MS analyses)

Identification of Na,K-ATPase α and β-subunit composition in the nervous tissue (2D-gel electrophoresis)

To establish, which subunits occur in the nervous tissue and form complexes, we analyzed different samples from 2D-gel electrophoretic purified and resolved α1 and β-NKA isoforms in the form of gel pieces cut out from the second, horizontal dimension gel (α1- band gel sample designated as I–IV, β-band gel samples designated as i–iv) and submitted them to mass spectrometric analyses (LC–MS/MS). The analysis was repeated and we here refer to the samples of the two 2D-gel runs as ones from gel 1 and gel 2. Due to slight voltage fluctuations the two gels were not congruent and the α1 and β-bands of each gel were therefore analyzed separately. For LC–MS/MS analyses, we chose gel slices that were widely separated on the gels, since we expected different subunit combinations to be located at different areas on the gels and be more separated from one another. In contrast, we found αB and αC in each α1-band gel samples (I-IV) and β2 and β3 in each β-band gel samples (i–iv) indicating that the nervous tissue consists mainly of the four subunits and that a close linkage between them is likely (Fig. 1c, d).

Our proteomic analyses identified 7–14 and 14–20 unique peptides for αB and αC across the gel samples, respectively. These high numbers of unique peptides and the high sequence coverage make the identification of both α1-subunits highly reliable. Conversely, we identified a maximum of four unique peptides for αA in one of the samples, giving less support for the identification of the subunit. Additionally, the existence of β2 and β3 in the nervous tissue was strongly supported by the detection of high numbers of unique peptides and thereby high sequence coverages (Tables S2 and S3).

For further analyses, we focused on the unique PSMs, as they are proportional to the protein abundances (Madsen et al. 2015). The analyses of both gels showed higher abundances of αC (compared to αB) and β3 (compared to β2) across the samples, represented by their unique PSMs. The abundances of the detected NKA subunits within the three and four analyzed samples showed a unique distribution pattern for each of the four subunits across the samples (Fig. 1c, d). In gel 1, the highest numbers of identified unique PSMs were found in sample I for both alphas and sample ii for both betas (Fig. 1c), and in the second gel, the highest numbers of identified unique PSMs were found in sample II for αC, in III for αB and sample ii for β3 and iii for both betas (Fig. 1d). The results reflect the protein migration through the first-dimensional gel (vertical run, Fig. 1a, b), indicating complexes with different weights based on different protein aggregation states, varying numbers of complex-forming subunits, different glycosylation states of the β-subunits or diversity in complex compositions (containing other membrane-bound proteins or lipids that were not included in our study).

Since αB, αC, and β2, β3 were found together in each of the distinct gel samples (α1-subunits in I–IV, β-subunits in i–iv samples), we wanted to compare the strength of support for their presence. To do this, we statistically tested whether the three α1 and the two β-subunits found in one gel differed significantly from one another with regard to the number of unique peptides, sequence coverage, and unique PSMs.

We found that due to low abundances in both gels, significantly less unique peptides, PSMs, and sequence coverage were identified for αA compared to αB and αC. Furthermore, αC showed the highest numbers of unique peptides, unique PSMs, and highest sequence coverage compared to αA and αB in both gels (Fig. 1e, f). Except for sequence coverage, these differences were identified as significant solely in samples of gel 2 for both comparisons (αA–αC, αA–αB; Table S4). The two β-subunits showed no significant differences in both gels, but β3 has higher numbers of unique peptides and unique PSMs as well as a higher sequence coverage in comparison to β2. A simultaneously used method named label-free quantification (MaxQuant LFQ) enabled us to compare relative quantities of one certain subunit across the samples. The results supported our previous findings. The abundances of all four subunits decreased from sample II to III (gel 1) (Fig. S5a). However, αB and β2 have their highest abundances in sample III/iii and αC and β3 in sample II/ii and III/iii (gel 2) (Fig. S5b). The latter result underpins the hypothesis of αC and β3 being a complex-forming unit since they occur at high abundances in corresponding samples.

### IP with magnetic beads

#### Enrichment and identification of Na,K-ATPase subunit complexes via magnetic bead IP

**Fig. 2:**
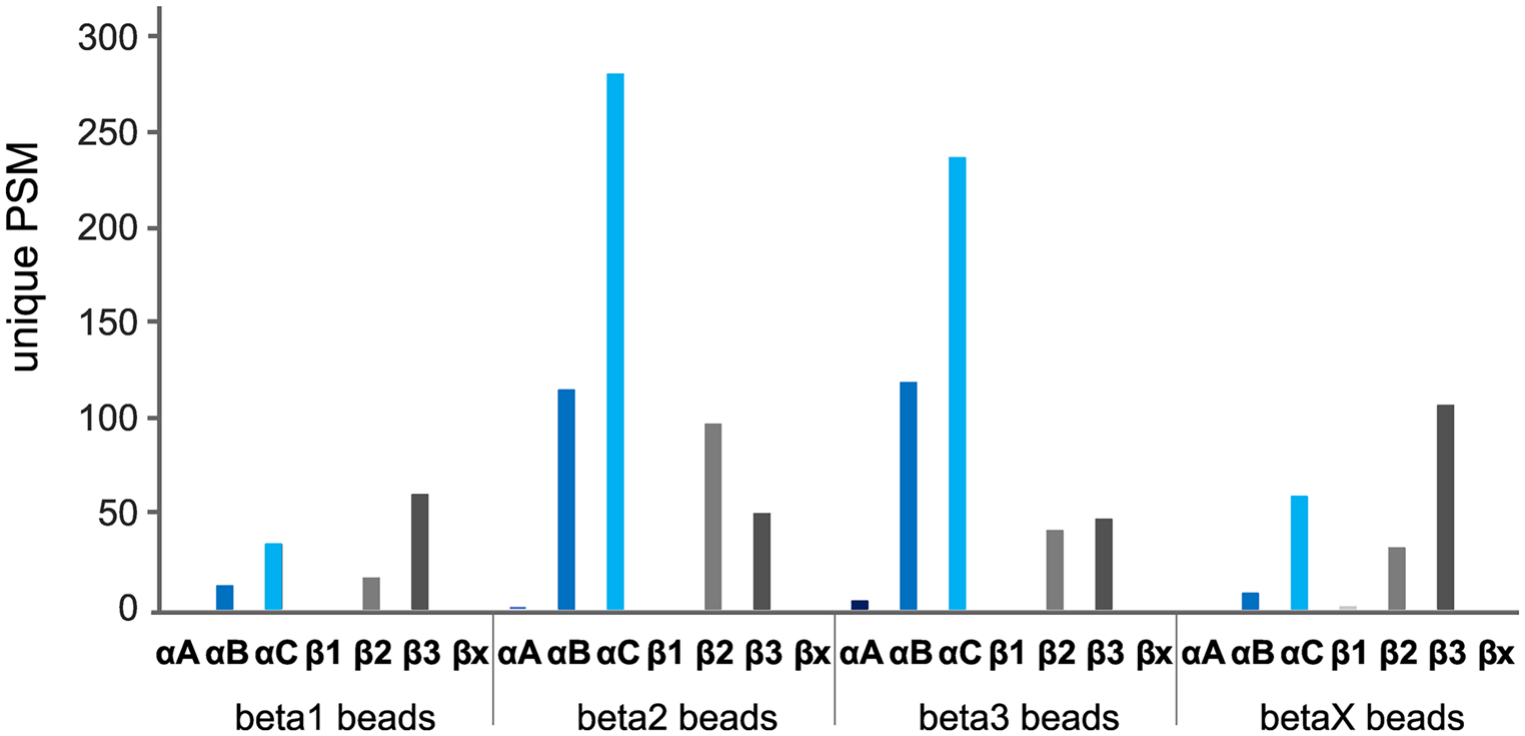
Analyses of unique PSMs originating from different subunits of the NKA (αA, αB, αC, β1, β2, β3) from nervous tissue samples of *O. fasciatus.* Data from LC–MS/MS analyses of eluates from immunoprecipitation with magnetic beads coupled with β-specific antibodies (β1, β2, β3, and βx respectively). Identified unique peptide-spectrum matches (unique PSMs) were plotted for every magnetic bead eluate immunoprecipitated with one specific β-antibody (beta1 beads, beta2 beads, beta3 beads, and betaX beads, respectively) for each detected NKA subunit

This technique identified a higher number of unique PSMs than revealed through 2D-gel electrophoresis and was thus more successful at extracting the protein complexes (Fig. 2). With the IP approach we expected to enrich just the β-subunit the specific antibody coupled to the magnetic beads was targeted on together with the associated α1-subunit. In contrast, we once again found αB, αC, β2, and β3 in all eluates independent of the antibody the beads were crosslinked with. We were also able to identify again αC and β3 as the subunits with the highest abundances across all eluates (except for the eluate of β2-antibody crosslinked beads, where β2 was most abundant). The number of unique PSMs of αA and β1 was negligible. Interactions between the four subunits, such as forming tetrameric or homo-dimeric structures, are highly possible and supported by our data since the highest unique PSMs for αB and αC could be found in the eluates of β2 and β3-antibody crosslinked beads in company with both β-subunits (Fig. 2).

Furthermore, LC–MS/MS analyses resulted in similar amounts of unique peptides and coverage proportions for all four subunits compared to the data gained by the 2D-gel electrophoresis approach (Table 1). We also found very few unique peptides for αA and β1 resulting in a low sequence coverage. High amounts of unique peptides could be again detected for αC, αB, β2, and β3, having a high sequence coverage across the eluates. Since we successfully purified the NKA subunits by using magnetic beads coupled to β1 and βx-specific antibodies, these subunits must be present although we could not extract the latter subunit with this approach. These data suggest that β1 and βx are present but to a lower extent and could therefore not be sufficiently detected by LC–MS/MS.

**Table 1:** Identification of NKA subunits after immunoprecipitation with magnetic beads coupled to β-specific antibodies. Counts of unique peptides, total peptides (sum of unique and shared peptides) and the subsequent sequence coverage were identified in each IP sample for each of the six subunits

β-specific antibody coupled to magnetic beads	Subunit detected	Unique peptides	Total peptides (unique and shared)	Sequence coverage [%]
β1	αA	0	16	17.3
	αB	6	31	36
	αC	12	34	39
	β1	0	0	0
	β2	8	8	24.8
	β3	10	10	42.7
β2	αA	1	22	23.3
	αB	15	50	52
	αC	23	55	54.4
	β1	0	0	0
	β2	16	16	40.4
	β3	8	8	31.8
β3	αA	3	21	24.5
	αB	14	47	50.6
	αC	21	49	54.1
	β1	0	0	0
	β2	10	10	32.6
	β3	8	8	37.9
βx	αA	0	15	17.4
	αB	5	32	40.3
	αC	14	37	46.2
	β1	1	1	4
	β2	10	10	29.8
	β3	9	9	42.4

### IHC of nervous tissue

Immunohistochemistry of intact nervous tissue of adult female milkweed bugs visualized the presence of all four β-subunits, yet in different tissue structures. For the α1-subunits, a similar resolution is not possible since the sequences are too similar to raise subunit-specific antibodies.

**Fig. 3:**
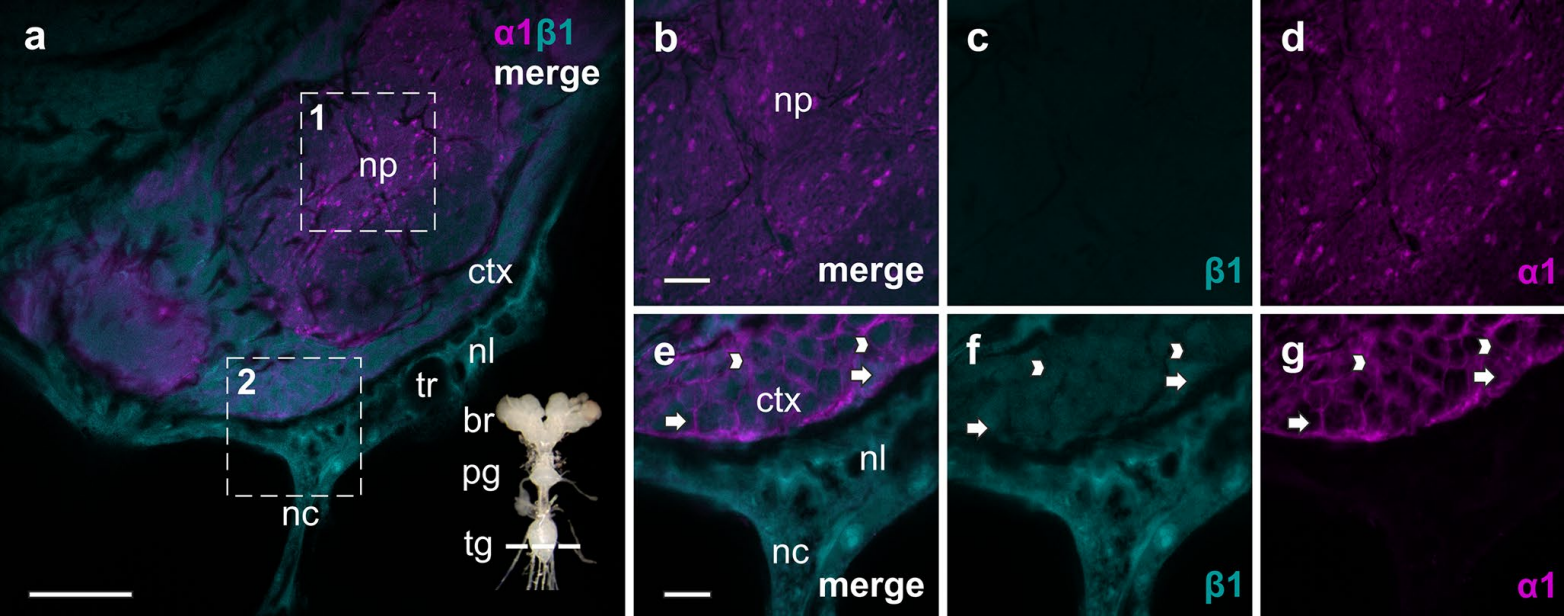
Distribution of the NKA subunits α1 (magenta) and β1 (cyan) in the fused metathoracic-abdominal part of the thoracic ganglion (tg). **b–d** In the neuropil (np) only α1 is highly abundant (magnified view from 1). **e–g** In the cortex (ctx) both subunits occur: α1 and β1 are present in neurons (arrows) and glial cells (arrowheads). β1 is also distributed in the neural lamella (nl) surrounding the ganglion, as well as the ganglion-entering nerve cord (nc) (magnified view from 2). The neural lamella is penetrated by trachea (tr). The IHC images are slightly modified: background was subtracted equally for both subunits using the “rolling ball” algorithm in imageJ. Scale bars shown in µm (merged overview image (**a**): 50 µm, magnification images (**b**–**g**): 12.5 µm). The nervous tissue image in the bottom right corner in **a** depicts the cutting plane for orientation: brain (br)*,* prothoracic ganglion (pg), thoracic ganglion (tg)

**Fig. 4:**
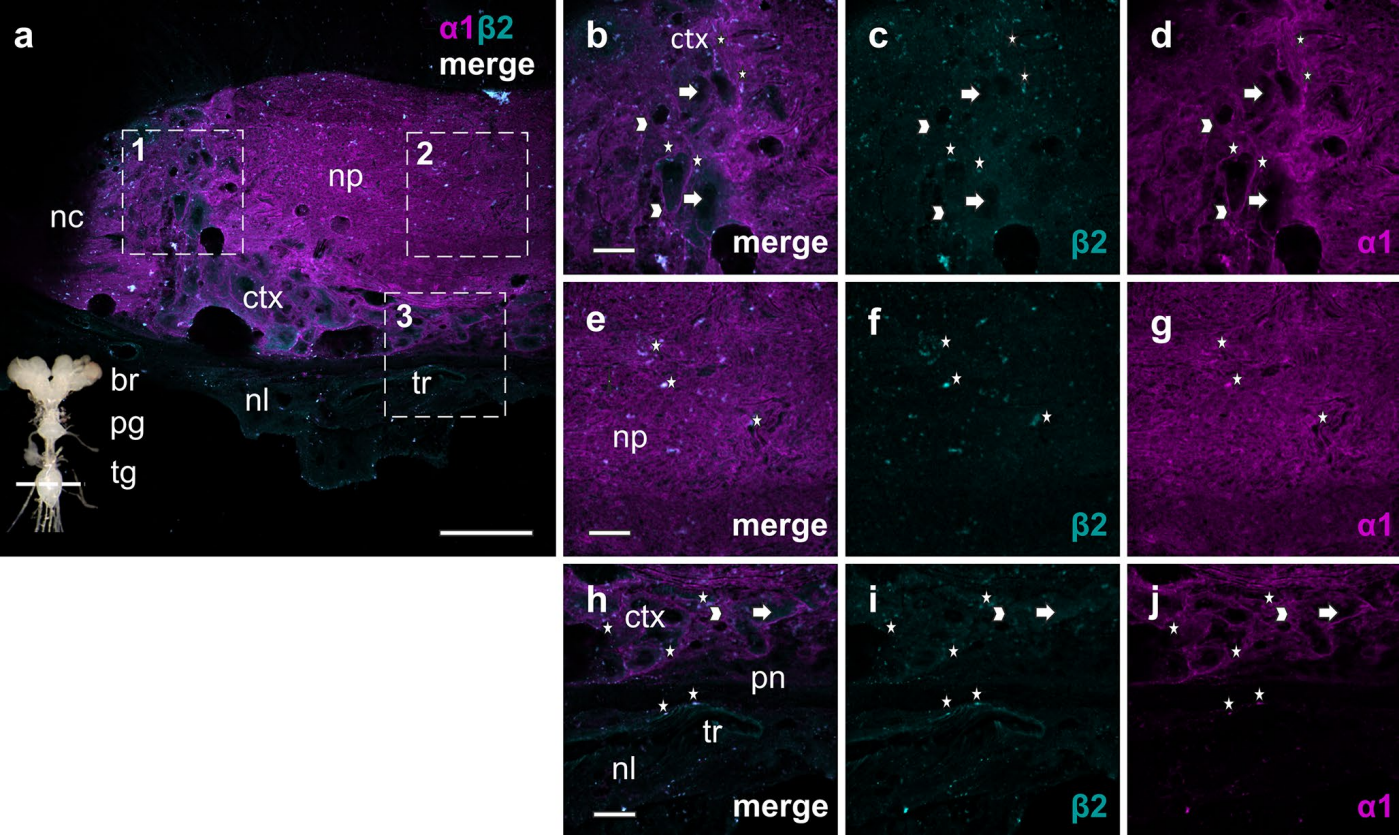
Distribution of the NKA subunits α1 (magenta) and β2 (cyan) in the mesothoracic part of the thoracic ganglion (tg) (dotted line through thoracic ganglion bottom left corner in **a)**. **a–g** In the axons of the neuropil (np) and nerve cords (nc) **h–j** as well as in the neuron (arrows) and glia cells (arrowheads) in the cortex (ctx) α1 and β2 are highly abundant as shown in the magnified view from 1 (**b–d**), 2 (**e–g**), and 3 (**h–j**). White asterisks show a selection of α1/ β2 colocalizations in the neuropil, cortex, and tracheal cells (tr). The IHC images are slightly modified: background was subtracted equally for both subunits using the “rolling ball” algorithm in imageJ. Scale bars shown in µm (merged overview image (**a**): 50 µm, magnification images (**b–j**): 12.5 µm)

**Fig. 5:**
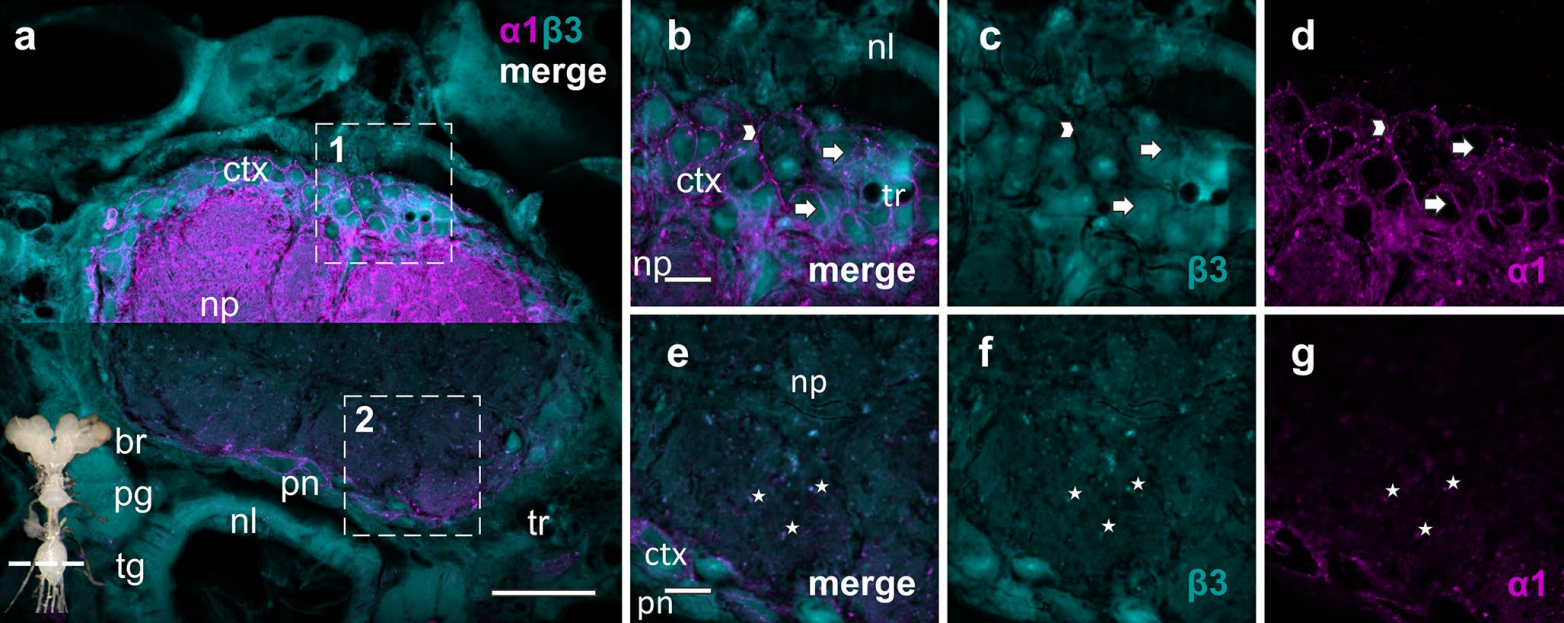
Distribution of the NKA subunits α1 (magenta) and β3 (cyan) across the metathoracic part of the thoracic ganglion (tg) (dotted line through thoracic ganglion bottom left corner). The merged overview image **a** is an assembly of two different focal planes. The α1-subunits are highly abundant in the neuropil (np) as well as in the neurons (arrows) and glial cells (arrowhead) in the cortex (ctx) but not in the neural lamella (nl) and less in the perineurium (pn) as shown in the magnified view from 1 (**b–d**) and 2 (**e–g**). β3 occurs in the neural lamella, perineurium, cell bodies across the cortex (ctx) and is also present in the neuropil where it is colocalized with α1-subunits (examples are highlighted with asterisks **e–g**). The IHC images are slightly modified: background was subtracted equally for both subunits using the “rolling ball” algorithm in ImageJ. Scale bars shown in µm (overview image (**a**): 50 µm, magnification image (**b–g**): 12.5 µm)

**Fig. 6:**
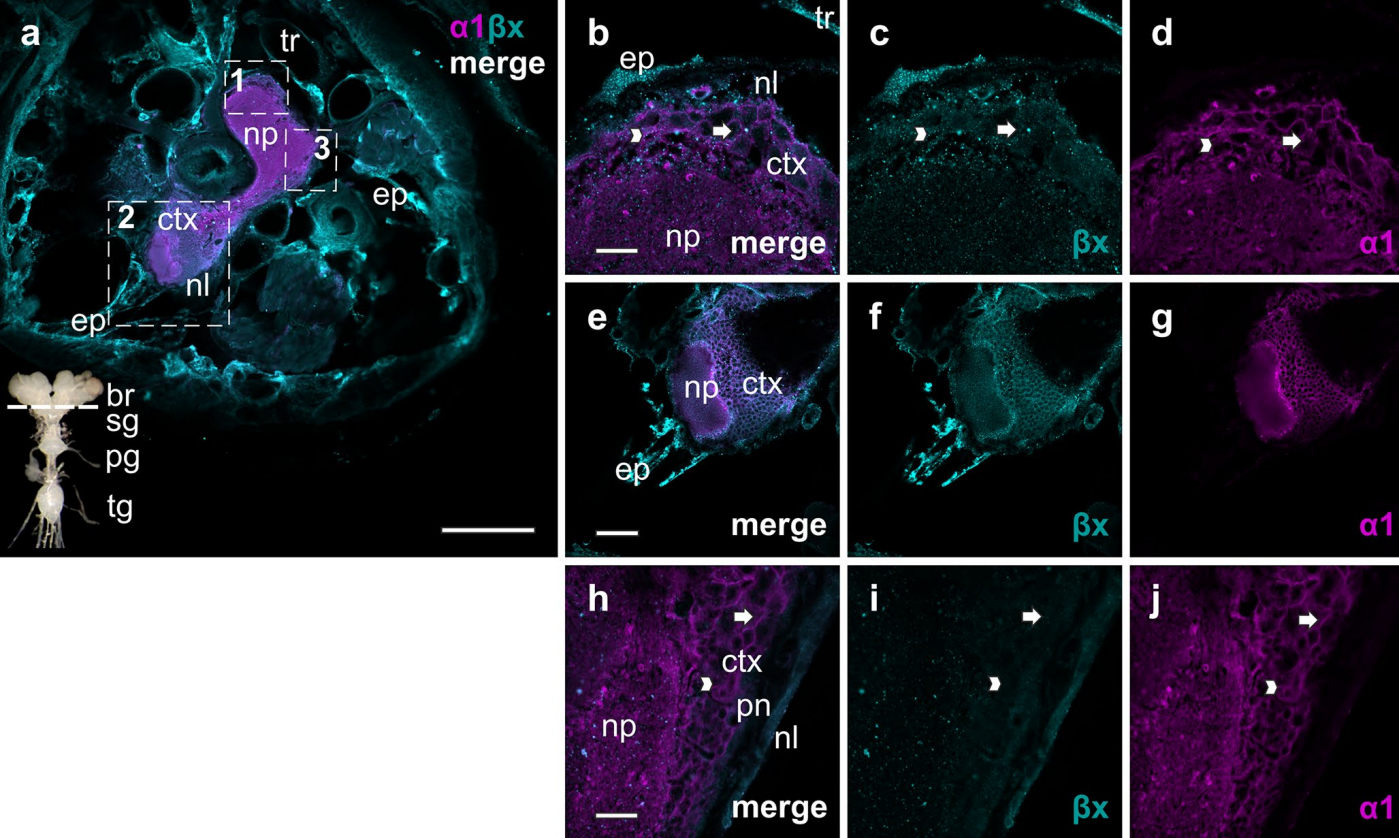
Distribution of the NKA subunits α1 (magenta) and βx (cyan) across the subesophageal ganglion (sg) (dotted line: cross section through subesophageal ganglion bottom left corner). The α1-subunits are highly abundant in the neuropil (np), in neurons (arrow) and glial cells (arrowhead) in the cortex (ctx) as shown in the magnified view from 1 (**b–d**), 2 (**e–g**), and 3 (**h–j**). βx appears in distinct spots all over the neuropil and cortex (magnified view from 1 to 3), the perineurium (pn), and neural lamella (nl) (magnified view from 3). The grainy structure is most pronounced in the epithelia (ep) enclosing all organs throughout the body (overview picture of the head (**a**) and magnified view from 2). The IHC images are slightly modified: background was subtracted using the “rolling ball” algorithm in ImageJ. Scale bars are shown in µm (overview image (**a**): 100 µm, magnification images **b–d** and **h–j**: 16.7 µm, **e–g**: 50 µm)

The nervous tissue of *O. fasciatus* is composed of the brain, subesophageal ganglion, prothoracic and thoracic ganglion (fused meso, meta, and abdominal ganglion) linked with connectives (see inset in Figs. 3–6). The neuropil is located in the center of the ganglia and brain. It is surrounded by the cortex, and this in turn is enclosed by the nerve sheath built from the perineurium and the neural lamella (Johansson 1957). In *Drosophila melanogaster* five types of glial cells were identified varying in their shape and localization across the nervous tissue: in the neuropil sheath-building and astrocyte-like glial cells were found, the cortex is packed with cortex glia and neurons, and in the outer layers perineural and subperineural glial cells were identified (Awasaki et al. 2008). In all these parts of the nervous system of *O. fasciatus*, the NKA is widely distributed. The α1-subunits were predominantly present in the neuropil with a relatively homogenous distribution, whereas also highly concentrated spots could be found all over the neuropil (Figs. 3–6). Additionally, the α1-subunits were present in the neurons and glial cells in the cortex to a similar extent. In the perineurium, a layer girding the cortex, which is composed of flat glial cells, less α1-subunits were detected, and none was identified in the neural lamella (Fig. 3). All over the cortex, β1 was identified in neurons and glial cells, where it was colocalized with α1 in the cell periphery. The β-subunit was not or very little expressed in the neuropil but was mainly identified in the neural lamella with a homogenous and diffuse distribution, surrounding the ganglia, brain, and the nerve cords (Fig. 3). β2 was abundant in all the structures of the ganglia and brain. The distribution pattern differed from that of β1 by being more concentrated and punctual. In the neuropil and cortex cells, β2 was colocalized with α1-subunits, as well as in tracheal cells that are located in the neural lamella (Fig. 4). β3 was diffusely and homogenously distributed all over the nervous tissue. In addition, the β3-subunit appeared at concentrated spots in the neuropil colocalizing with α1. It was, as well as β2, also abundant in glial cells in the cortex, but mainly in neurons and like β1, it was abundant in the neural lamella (Fig. 5). Similar to the other β-subunits, βx was present in the neuropil, cortex, and the neural lamella. In comparison to β3, the distribution pattern of βx differed in the neuronal cells in the way that the subunit seems to be more abundant in the periphery of the cells than in the center. Like β2, βx was punctually concentrated and distributed all over the tissue. When viewing the merged images of Fig. 6, βx was not colocalized with α1-subunits since no color-overlay was recognizable. However, βx was as sole β-subunit abundant in a membranous layer (epithelium) surrounding organs including the nervous tissue (Fig. 6).

## Discussion

Amino acid substitutions in the CG-binding site of the NKA may counteract inhibition by CGs. While such substitutions can alter the resistance of the enzyme to CGs, they can also alter its kinetic properties, and often, enzymes achieve resistance to CGs at the cost of reduced ion pumping function (Dalla et al. 2017; Herbertz et al. 2021). The trade-offs between resistance and ion pumping activity favor the evolution of paralogs that can compensate for such negative pleiotropic effects (Dalla and Dobler 2016; Mohammadi et al. 2021). *O. fasciatus* represents a prime example for this: gene duplications lead to a total of four α1-subunits with various substitutions in the CG-binding site (Zhen et al. 2012; Yang et al. 2019) and with variation in their CG-resistance and overall NKA activity (Herbertz et al. 2021). In addition, the combination with four β-subunits further modifies the properties of the enzyme (Herbertz et al. 2021). To further unravel how trade-offs and compensations might fit together in these enzymes, we analyzed the presence and abundance of possible protein complexes in the nervous tissue of adult large milkweed bugs.

### Distribution of NKA α1-subunits in tissues o*f O. fasciatus*

Our LC–MS/MS data revealed that αB and αC are the main NKA α-isoforms in the nervous tissue of *O. fasciatus*. These two α1-subunits differ strongly in their kinetic and physical properties: αB is highly resistant against CGs while having a significantly lower ion pumping activity than αC (Herbertz et al. 2021). Yet, even though αC is more easily inhibited than αB, it is still strongly resistant against CGs — even more so than the NKA of the famous monarch butterfly (Petschenka et al. 2013, 2018). The occurrence of both α1-subunits apparently provides the nervous tissue of *O. fasciatus* with both high ion-pumping capacity (αC) and enhanced resistance against CGs dissolved in the hemolymph (αB).

Our proteomic results match with mRNA abundances determined by quantitative real-time PCR, which detected higher expression levels of the *αC* gene compared to *αB* in the nervous tissue in *O. fasciatus* (Zhen et al. 2012; Lohr et al. 2017). Only small amounts of αA could be detected in the nervous tissue by our LC–MS/MS analyses. Its abundance seems to be negligible since less unique peptides of αA were found than for αB and αC, indicating that αA plays only a minor role in the nervous tissue of *O. fasciatus.* Quantitative real-time PCR data previously suggested that the two most CG-resistant subunits *αA* and *αB* were very abundant in the gut and in the Malpighian tubules (Lohr et al. 2017), which are involved in the metabolism of cardenolides (Meredith et al. 1984). The tissue-specific expression profiles of the different α1-isoforms fit with their CG resistance when considering that the gut and Malpighian tubules are directly exposed to CGs, while the nervous tissue is protected by the perineurium and a massive neural lamella. However, it is unclear to what extent CGs are still able to enter through these protective barriers. In the well protected nervous tissue, a fast transfer of information and a quick restoring of resting potentials can apparently best be achieved by α1C, the highly active, yet less resistant NKA α1-subunit.

Our IHC images underline the predominance of the NKA in the nervous tissue: the neuropil, and the surrounding glial cells and neurons in the cortex are completely permeated with α1-subunits. Sporadically distributed accumulations of α1-subunits are visible all over the neuropil (recognizable as light magenta-colored dots), which might possibly correspond to synapses. Conversely, barely detectable fluorescence signals suggest that α1-subunits are almost totally absent from the perineurium and the neural lamella.

The assembly of α with β was intensively studied by Geering and coworkers and clearly supported the dependence of the α on the β-subunit for membrane integration and functionality (Geering 1990, 2001; McDonough et al. 1990; Geering et al. 1996; Hasler et al. 1998a, b). Histological studies of *Drosophila’s* brain also confirmed the presence of both NKA-subunits (Baumann et al. 2010). In stark contrast to our current understanding, it seems as if the α1-subunits are to some extent present without an associated β-subunit. Since the α1-subunits cannot be discriminated by antibodies, it is impossible to determine whether this concerns αB or αC, and further investigations are needed to validate these findings.

### Distribution of NKA β-subunits in tissues of *O. fasciatus*

While the β-subunit was originally attributed a chaperone-like role for the correct membrane integration of the functional NKA (Geering 2001), recent analyses revealed additional roles ranging from modulation of the kinetic behavior of the NKA to morphogenic functions (Geering 2001; Blanco 2005; Paul et al. 2007; Dalla et al. 2017)*.* The mammalian NKA β2-subunit, for example, is also known as the adhesion molecule on glia (AMOG) contributing to the adhesion between neurons and astrocytes and instigating granule cell migration in the cerebellum (Gloor et al. 1990).

Much less is known about differential expression of β-paralogs in insects compared to mammals. In *D. melanogaster* three β-paralogs (Nrv1, Nrv2 and Nrv3) are known, of which Nrv1 was mainly found in muscle cells and the other two are found in the nervous tissue: Nrv2 is predominantly expressed in epithelia, in neurons in the brain and the optic lobes, while Nrv3 is highly abundant in the retina, mechanosensory neurons, chemosensory cells, and somewhat less abundant in the brain (Sun et al. 1998; Paul et al. 2007; Baumann et al. 2010; Roy et al. 2013). Based on protein sequence similarity (Stothard 2000), three of the β-subunits of *O. fasciatus* (β1–β3) can be homologized with their counterparts in *D. melanogaster* (Herbertz et al. 2021; Table S5). The fourth β-subunit of *O. fasciatus*, βx, lacks the cytosolic N-terminal domain, which influences the secondary structure and thus indirectly the ion-transport function (Hasler et al. 1998b). It was thus unclear whether this gene is at all translated into a functional protein.

Similar to the situation in *D. melanogaster* the LC–MS/MS analyses revealed β2 and β3 as the predominant β-subunits in the nervous system of *O. fasciatus*. The IHC analyses further supported the joint occurrence of β2 and β3 in the neuropil and showed a characteristic pattern for each of them in other structures of the ganglion. The β2-subunit is present throughout the neuropil and cortex, and specifically located in neurons as well as glial cells. In the neural lamella β2 is mainly present in tracheal cells. In contrast, β3 is diffusively spread all over the neural sheath and can be found in the center and periphery of glial and neuronal cell bodies. Part of the β3 molecules are seemingly not integrated into the membranes but appear to be caught in the cytosol waiting for an assembly and targeting to the membrane. In any case, the β3-subunits seem to be detached from the α1-subunits that are clearly not present in these areas, as no fluorescence signals of α1 were detectable.

Another striking observation is the lack of contact to an α1-subunit of those β3-subunits present in the cell bodies and the neural sheath enclosing the whole nervous tissue. This suggests that β3 may homodimerize with other β3-subunits and be involved in cell adhesion. The ability of NKA β-subunits to form homodimers has been extensively demonstrated for mammalian epithelial junctions in Madin-Darby Canine Kidney (MDCK) cells (reviewed in Vagin et al. 2012). Here the β-subunits of α1β1-dimers form intercellular assemblies, which are stabilized by N-glycans and a specific outward facing ten amino acid sequence, present on the extracellular loop. A junctional activity of β-subunits has also been demonstrated for Nrv2 in *D. melanogaster* (Paul et al. 2003, 2007; see below) and is just as well conceivable for the isolated β3-subunits of *O. fasciatus*.

Of the remaining two β-subunits of *O. fasciatus*, β1 could barely be detected by LC–MS/MS analysis, yet IHC gave a better picture of its abundance. This subunit is least represented in the nervous tissue and shows a diffuse distribution as it was also described for Nrv1 (Sun et al. 1998; Baumann et al. 2010). β1 seems to be totally absent from the neuropil of the brain and ganglia. The low abundance of β1 in the nervous system fits with its single specific peptide detected by LC–MS/MS, indicating its presence at the detection limit.

The fourth β-subunit of *O. fasciatus*, βx, lacks the cytosolic N-terminal domain, which influences the secondary structure and thus indirectly the ion-transport function (Hasler et al. 1998b). It was thus previously unclear whether this gene is at all translated into a functional protein. Although our LC–MS/MS analyses were unable to detect βx in nervous tissue samples, we were able to confirm its presence as a functional protein. However, βx antibodies could be used to precipitate and identify other subunits. The IHC analyses clarified the situation and clearly supported the presence of βx showing that the subunit is mostly present in the epithelia throughout the large milkweed bugs’ body, but is also present in the neuropil, the cortex, and the neural sheath.

Methodological problems of the LC–MS/MS analyses might in principle explain the failure to identify the truncated βx-subunit: peptides can only be reliably detected between a size range of 500 to 2000 Da and in dependence of their ionization capacity. Peptides outside this range stay often unnoticed, and sequences with free cysteine residues are seldomly spotted (Fricker 2015). In silico tryptic digest predicted 9 out of a total of 20 peptides within the decisive size range, excluding those with a cysteine residue. To evaluate potential methodological problems, we submitted a highly concentrated sample of heterologously expressed βx (combined with αB) to LC–MS/MS analysis. All in silico predicted peptides could be identified, no matter their size or cysteine content. We hence suggest that high glycosylation levels lead to an incomplete tryptic digest beforehand or that the presence and abundance of other proteins/peptides in the nervous tissue samples masked the target peptides during mass spectrometric analyses and prevented a detection of βx.

### Associations of NKA α and β-subunits in the nervous tissue

The second aim of our study was to determine and locate the α1/β-combinations present in the nervous tissue. Our previous in vitro expression studies support that they all form functional enzymes (Herbertz et al. 2021), as has also been shown in other systems (Lemas et al. 1994; Mobasheri et al. 2000). Despite the array of methodological approaches used no simple answers emerge. As the LC–MS/MS data support, two α1-subunits, αB and αC, and two β-subunits, β2 and β3, predominate and are supported by large numbers of PSMs and strong LFQ intensities. Although quantitative comparisons of LC–MS/MS data of different proteins are difficult due to methodological limitations (reviewed by Xie et al. 2011), the sum of PSMs as well as mRNA levels (analyzed by Lohr et al. (2017)) suggest that αC is the most abundant α1-subunit. Likewise the β-subunits, β2, and β3 share similar mRNA expression levels (Herbertz et al. in prep.) matching our protein level data.

While an analysis of the PSM abundances in different gel slices suggested a predominant association of αB with β2 and αC with β3, these associations are not exclusive. The 2D-gel electrophoresis using non-denaturing conditions in the first dimension still yielded samples comprising all four subunits plus small amounts of αA. Likewise immunoprecipitation with β-specific antibodies yielded both αB and αC as well as additional β-subunits. Both lines of evidence argue for larger mixed associations of α1/β-combinations in addition to the postulated binary complexes. Previous studies on mammal renal tissues and insect cell culture experiments support higher order or homodimeric associations of NKA subunits (Taniguchi et al. 2001; Laughery et al. 2004).

The fine-scale resolution of the IHC-images shows that the β2-subunit is colocalized with α1-subunits in all nervous tissue structures (visualized as overlapping signals in the same focal plane). Although we cannot discriminate the α1-subunits by IHC, we can generate plausible hypotheses based on their known properties (Herbertz et al. 2021). We expect that αCβ2 as a highly active NKA should be the dominant NKA complex in the neuropil where numerous axons are bundled and fast information transfer should be essential. The neuropil is surrounded by different layers: the cortex, followed by the perineurium (blood–brain barrier) and finally the neural lamella. The latter two form the neural sheath, which protects the brain and ganglia from harmful compounds in the hemolymph. However, the ion transfer from the hemolymph into the nervous tissue happens through NKAs sitting in the perineurium, therefore exposure to toxins could not be excluded, additionally it is not known to what extent CGs leak into the nervous tissue by passing septate and gap junctions located in the neural sheath (Treherne and Schofield 1981; Hou et al. 2014). We would expect that αBβ2 as well as αCβ2 combinations are present depending on the localization within the cell, the concomitant exposition and cell function. In more exposed cells located in the perineurium or in vicinity to the neural sheath, we would expect a higher proportion of αBβ2 complexes, as a vastly more resistant form of the NKA.

Besides forming functional ion pumping NKAs, it is highly likely that β2 together with an α1-subunit is involved in septate junction formation. As described above, α1β1-dimers are an essential part of junctional complexes in mammals (reviewed in Vagin et al. 2012), and likewise complexes of Nrv2 and α1 induces the formation of septate junctions in *D. melanogaster*. These complexes influence tracheal development, length and diameter, as well as epithelial junction functionality (Paul et al. 2003, 2007). Septate junctions in the subperineural layer (situated right under the perineurium), formed by specialized glial cells, are part of the blood–brain barrier in the fruit fly’s brain preventing leakage of hemolymph into the nervous tissue (Limmer et al. 2014). Since NKA-mediated junction formation seems to represent an evolutionarily conserved process and requires no pumping activity of the α-subunit (Paul et al. 2007), we expect similar structures in the nervous tissue of *O. fasciatus*, most likely consisting of αBβ2 complexes.

In some regions of the neuropil, superposition of stainings suggest a colocalization of α1 and β3. Similar to the situation for β2, a combination with αB or αC is possible. However, our LC–MS/MS data gave the highest mean PSM counts and similar PSM distribution across the 2D-gel samples for αC and β3, thus complexes of these two subunits appear most likely. In addition, this combination showed the highest ion pumping activity of all our heterologously expressed α1/β constructs (Herbertz et al. 2021) which should be favorable in the nervous tissue.

In contrast to β2 and β3, no local overlaps for βx and α1 could be observed in any of the nervous tissue structures. βx seems to stand alone to fulfill so far unknown functions. Its localization, distribution pattern and the absence of an associated α1-subunit once more allow for speculations about a possible role in the formation of cell–cell contacts, yet further functional data are needed to better understand this non-canonical β-subunit.

Overall, our results provide the first insights into the distribution and potential functions of Na,K-ATP α1 and β-subunits in the nervous tissue of *O. fasciatus*. The different properties of these subunits and the resulting NKA complexes obviously create new opportunities to accommodate specific functional needs in the toxin-exposed tissues of *O. fasciatus*.

## Supplementary Information

Below is the link to the electronic supplementary material.

Supplementary file1 (PDF 1228 KB)

Supplementary file2 (PDF 8121 KB)
