# Supplementary material for: Na,K-ATPase α1 and β-subunits show distinct localizations in the nervous tissue of the large milkweed bug
Source: Cell Tissue Res. 2022 Mar 25;388(3):503–19. doi: 10.1007/s00441-022-03580-6 (PMC9110512; doi:10.1007/s00441-022-03580-6)
Supplement: Supplementary file 1 — Supplementary file1 (PDF 1228 KB) [file 441_2022_3580_MOESM1_ESM.pdf]

Supplemental Information

Tables S1-S5

Na,K-ATPase α1 and β-subunits show distinct localizations in the nervous tissue of the large milkweed bug

Cell and tissue research

Marlena Herbertz[1*], Sönke Harder[2], Hartmut Schlüter[2], Christian Lohr[3], Susanne Dobler[1]

[1]Institute of Zoology, Molecular Evolutionary Biology, Universität Hamburg, 20146 Hamburg, Germany

[2]Institute of Clinical Chemistry and Laboratory Medicine, University Medical Center Hamburg-Eppendorf, 20246 Hamburg, Germany

[3]Institute of Zoology, Neurophysiology, Universität Hamburg, 20146 Hamburg, Germany

Corresponding author: Marlena Herbertz

E-mail: marlena-winter@uni-hamburg.de

Table S1: Molecular weights of all three α1 and four β-subunits of the NKA of *O. fasciatus.*

Na,K-ATPase subunit	Molecular weight [kDa]
αA	111.588
αB	109.085
αC	110.047
β1	38.366
β2	37.428
β3	36.636
βx	27.621

Table S2: With LC-MS/MS identified unique (bold font) and shared peptides (italic font) from the three α1 and four β-subunits.

NKA-subunit	2D-Gel gel 1	2D-Gel gel 2	magnetic bead IP
αA	**NSIIHQGmR**<br>*AAVPDAVAK*<br>*FGDRIPADIR*<br>*FVGLISmIDPPR*<br>*GFKVDNSSLTGESEPQSR*<br>*IATLcNR*<br>*KVcEIPFNSTNK*<br>*LIFDNLK*	**ADIGIAMGISGSDVSK**<br>**AEFKPGQEGVPILR**<br>**FETNPETGLSHDK**<br>**IAGLASGLDTGSTPIAR**<br>**SVGIISEGNETVEDIAHR**<br>*AAVPDAVAK*	**AVNDLQDSYGGQEWTYK**<br>**RFETNPETGLSHDK**<br>**SVGIISEGNETVEDIAHR**<br>*AAVPDAVAK*<br>*FGDRIPADIR*<br>*FVGLISMIDPPR*<br>*GFKVDNSSLTGESEPQSR*<br>*IATLcNR*

	LIFDNLKK<br>*LIIVEGcQR*<br>*LNIPISEVNPR*<br>*mGAIVAVTGDGVNDSPALK*<br>*NLAFFSTNAVEGTAK*<br>*NLEAVETLGSTSTIcSDK*<br>*NLEAVETLGSTSTIcSDKTGTLTQNR*<br>*QAADmILLDDNFASIVTGVEEGR*<br>*TGTLTQNR*<br>*VcEIPFNSTNK*<br>*VDNSSLTGESEPQSR*<br>*VImVTGDHPITAK*<br>*YHTEIVFAR*<br>*YLmVmK*<br>*YQVSIHETEDPNDSR*	*FGDRIPADIR*<br>*FVGLISMIDPPR*<br>*GFKVDNSSLTGESEPQSR*<br>*IATLCNR*<br>*KVCEIPFNSTNK*<br>*LIFDNLK*<br>*LIFDNLKK*<br>*LIIVEGCQR*<br>*LNIPISEVNPR*<br>*MGAIVAVTGDGVNDSPALK*<br>*MGAIVAVTGDGVNDSPALKK*<br>*NLAFFSTNAVEGTAK*<br>*NLEAVETLGSTSTICSDK*<br>*NLEAVETLGSTSTICSDKTGTLTQNR*<br>*QAADMILLDDNFASIVTGVEEGR*<br>*SAGIKVIMVTGDHPITAK*<br>*VCEIPFNSTNK*<br>*VDNSSLTGESEPQSR*<br>*VIMVTGDHPITAK*<br>*YHTEIVFAR*<br>*YQVSIHETEDPNDSR*	*KVcEIPFNSTNK*<br>*LIFDNLK*<br>*LIFDNLKK*<br>*LIIVEGcQR*<br>*LNIPISEVNPR*<br>*MGAIVAVTGDGVNDSPALK*<br>*MGAIVAVTGDGVNDSPALKK*<br>*NLAFFSTnAVEGTAK*<br>*NLEAVETLGSTSTIcSDK*<br>*nLEAVETLGSTSTIcSDKTGTLTQNR*<br>*QAADMILLDDnFASIVTGVEEGR*<br>*VcEIPFNSTNK*<br>*VDnSSLTGESEPQSR*<br>*VIMVTGDHPITAK*<br>*YHTEIVFAR*<br>*YQVSIHETEDPNDSR*
αB	**AAVVHGSELR**<br>**AEEIVLGDVVEVK**<br>**AEFKPGQDGIPILK**<br>**AKELLER**<br>**AVNDLQDSYGQEWTYR**<br>**cmELALGDIVSIR**<br>**FGTNPETGLTHAK**<br>**FVGLISmIDPPR**<br>**GIEmTNDNPLETK**<br>**GVVIScGDR**<br>**ISLEELFQR**<br>**LNIPISEVNPR**<br>**NmVPQFAIAVR**<br>**NPFTDKLVNER**<br>**NPGGWLER**<br>**YQVSIHETEDPNDSR**<br>*AAVPDAVAK*<br>*ADIGVAmGISGSDVSK*<br>*cSTIFIGGK*<br>*DGPNALTPPK*<br>*DTSPEQLDEILR*<br>*EAFNNAYLELGGLGER*<br>*EKLLDEEmK*<br>*EVNGDASEAALLK*	**AAVVHGSELR**<br>**AEEIVLGDVVEVK**<br>**AEEIVLGDVVEVKFGDR**<br>**AEFKPGQDGIPILK**<br>**AVNDLQDSYGQEWTYR**<br>**CMELALGDIVSIR**<br>**FGTNPETGLTHAK**<br>**GIEMTNDNPLETK**<br>**GVVISCGDR**<br>**ISLEELFQR**<br>**NMVPQFAIAVR**<br>**NPFTDKLVNER**<br>**NPGGWLER**<br>**RNPGGWLER**<br>*AAVPDAVAK*<br>*ADIGVAMGISGSDVSK*<br>*AIAKSVGIISEGNETVEDIAQR*<br>*CSTIFIGGK*<br>*DGPNALTPPK*<br>*DTSPEQLDEILR*<br>*EAFNNAYLELGGLGER*	**AAVVHGSELR**<br>**AAVVHGSELRDTSPEQLDEILR**<br>**AEEIVLGDVVEVK**<br>**AEFKPGQDGIPILK**<br>**AVNDLqDSYGQEWTYR**<br>**cMELALGDIVSIR**<br>**ELLERDGPNALTPPK**<br>**FGTNPETGLTHAK**<br>**GIEMTNDNPLETK**<br>**ISLEELFQR**<br>**NMVPQFAIAVR**<br>**NPFTDKLVNER**<br>**NPGGWLER**<br>**REVnGDASEAALLK**<br>**RNPGGWLER**<br>*AAVPDAVAK*<br>*ADIGVAMGISGSDVSK*<br>*cSTIFIGGK*<br>*DGPNALTPPK*<br>*DTSPEQLDEILR*<br>*EAFNNAYLELGGLGER*<br>*EKLLDEEMK*<br>*EVnGDASEAALLK*

	FGDRIPADIR<br>*FVGLISmIDPPR*<br>*GFKVDNSSLTGESEPQSR*<br>*IAGLASGLDTGETPIAK*<br>*IATLcNR*<br>*KADIGVAmGISGSDVSK*<br>*KVcEIPFNSTNK*<br>*LIFDNLK*<br>*LIFDNLKK*<br>*LIIVEGcQR*<br>*LLDEEmK*<br>*LLDEEmKEAFNNAYLELGGLGER*<br>*LNIPISEVNPR*<br>*mGAIVAVTGDGVNDSPALK*<br>*mTVAHmWFDNQIIEADTTEDQSGVQYDR*<br>*NLAFFSTNAVEGTAK*<br>*NLEAVETLGSTSTIcSDK*<br>*NLEAVETLGSTSTIcSDKTGTLTQNR*<br>*NSIFHQGmR*<br>*QAADmILLDDNFASIVTGVEEGR*<br>*SVGIISEGNETVEDIAQR*<br>*TGTLTQNR*<br>*TTPEWVK*<br>*VcEIPFNSTNK*<br>*VDNSSLTGESEPQSR*<br>*VImVTGDHPITAK*<br>*YHTEIVFAR*<br>*YLmVmK*<br>*YQVSIHETEDPNDSR*	*EKLLDEEMK*<br>*EVNGDASEAALLK*<br>*FGDRIPADIR*<br>*FVGLISMIDPPR*<br>*GFKVDNSSLTGESEPQSR*<br>*IAGLASGLDTGETPIAK*<br>*IATLCNR*<br>*KADIGVAMGISGSDVSK*<br>*KVCEIPFNSTNK*<br>*LIFDNLK*<br>*LIFDNLKK*<br>*LIIVEGCQR*<br>*LLDEEMK*<br>*LLDEEMKEAFNNAYLELGGLGER*<br>*LNIPISEVNPR*<br>*MGAIVAVTGDGVNDSPALK*<br>*MGAIVAVTGDGVNDSPALKK*<br>*MTVAHMWFDNQIIEADTTEDQSGVQYDR*<br>*NLAFFSTNAVEGTAK*<br>*NLEAVETLGSTSTICSDK*<br>*NLEAVETLGSTSTICSDKTGTLTQNR*<br>*NSIFHQGMR*<br>*QAADMILLDDNFASIVTGVEEGR*<br>*SAGIKVIMVTGDHPITAK*<br>*SVGIISEGNETVEDIAQR*<br>*TTPEWVK*<br>*VCEIPFNSTNK*<br>*VDNSSLTGESEPQSR*<br>*VIMVTGDHPITAK*<br>*YHTEIVFAR*<br>*YQVSIHETEDPNDSR*	*FGDRIPADIR*<br>*FVGLISMIDPPR*<br>*GFKVDNSSLTGESEPQSR*<br>*IAGLASGLDTGETPIAK*<br>*IATLcNR*<br>*KADIGVAMGISGSDVSK*<br>*KVcEIPFNSTNK*<br>*LIFDNLK*<br>*LIFDNLKK*<br>*LIIVEGcQR*<br>*LLDEEMKEAFNNAYLELGGLGER*<br>*LNIPISEVNPR*<br>*MGAIVAVTGDGVNDSPALK*<br>*MGAIVAVTGDGVNDSPALKK*<br>*MTVAHMWFDNQIIEADTTEDQSGVQYDR*<br>*NLAFFSTnAVEGTAK*<br>*NLEAVETLGSTSTIcSDK*<br>*nLEAVETLGSTSTIcSDKTGTLTQNR*<br>*NSIFHQGMR*<br>*QAADMILLDDnFASIVTGVEEGR*<br>*RNSIFHQGMR*<br>*SVGIISEGNETVEDIAQR*<br>*VcEIPFNSTNK*<br>*VDnSSLTGESEPQSR*<br>*VIMVTGDHPITAK*<br>*YHTEIVFAR*<br>*YQVSIHETEDPNDSR*
αC	**AAVVHGTDLR**<br>**AEDIVLGDVVEVK**<br>**AEFKGGQEGVPILK**<br>**AVNDLLDSYGQEWTYQDR**<br>**cmELALGDVLSIR**<br>**FDcDDPNFPLSGLR**<br>**FSTHPDSGLTHAK**<br>**FVGLmSmIDPPR**<br>**FVGLMSMIDPPRAAVPDAVAKcR**<br>**GGQEGVPILK**<br>**GGQEGVPILKK**	**AAVVHGTDLR**<br>**AAVVHGTDLRDTSPEQLDEILR**<br>**AEDIVLGDVVEVK**<br>**AEFKGGQEGVPILK**<br>**AVNDLLDSYGQEWTYQDR**<br>**AVNDLLDSYGQEWTYQDRK**<br>**CMELALGDVLSIR**<br>**FDCDDPNFPLSGLR**<br>**FSTHPDSGLTHAK**<br>**FVGLMSMIDPPR**	**AAVVHGTDLR**<br>**AAVVHGTDLRDTSPEQLDEILR**<br>**AEDIVLGDVVEVK**<br>**AEFKGGQEGVPILK**<br>**AKENLERDGPNALTPPK**<br>**AVNDLLDSYGQEWTYQDR**<br>**cMELALGDVLSIR**<br>**ENLERDGPNALTPPK**<br>**FDcDDPNFPLSGLR**<br>**FSTHPDSGLTHAK**<br>**FVGLMSMIDPPR**

	**GVENTHENPLETK**<br>**GVVIScGDNTVmGR**<br>**ISVEELYQR**<br>**LNIPVSEVNPR**<br>**NmVPQFATVVR**<br>**NPGGWLEQETYY**<br>**RNPGGWLEQETYY**<br>**YQVSIHETEDPNDPR**<br>*AAVPDAVAK*<br>*ADIGVAmGISGSDVSK*<br>*cSTIFIGGK*<br>*DGPNALTPPK*<br>*DTSPEQLDEILR*<br>*EAFNNAYLELGGLGER*<br>*EKLLDEEmK*<br>*EVNGDASEAALLK*<br>*FGDRIPADIR*<br>*GFKVDNSSLTGESEPQSR*<br>*IAGLASGLDTGETPIAK*<br>*IATLcNR*<br>*KADIGVAmGISGSDVSK*<br>*KVcEIPFNSTNK*<br>*LIFDNLK*<br>*LIFDNLKK*<br>*LIIVEGcQR*<br>*LLDEEmK*<br>*LLDEEmKEAFNNAYLELGGLGER*<br>*mGAIVAVTGDGVNDSPALK*<br>*mTVAHmWFDNQIIEADTTEDQSGVQYDR*<br>*NLAFFSTNAVEGTAK*<br>*NLEAVETLGSTSTIcSDK*<br>*NLEAVETLGSTSTIcSDKTGTLTQNR*<br>*NSIFHQGmR*<br>*QAADmILLDDNFASIVTGVEEGR*<br>*SVGIISEGNETVEDIAQR*<br>*TGTLTQNR*<br>*TTPEWVK*<br>*VcEIPFNSTNK*<br>*VDNSSLTGESEPQSR*<br>*VImVTGDHPITAK*<br>*YHTEIVFAR*	**GDLDDLKQELDIDHHK**<br>**GGQEGVPILK**<br>**GGQEGVPILKK**<br>**GVENTHENPLETK**<br>**GVVISCGDNTVMGR**<br>**IMESFKNMVPQFATVVR**<br>**ISVEELYQR**<br>**KEVNGDASEAALLK**<br>**LNIPVSEVNPR**<br>**NMVPQFATVVR**<br>**NPGGWLEQETYY**<br>**RNPGGWLEQETYY**<br>**YQVSIHETEDPNDPR**<br>*AAVPDAVAK*<br>*ADIGVAMGISGSDVSK*<br>*AIAKSVGIISEGNETVEDIAQR*<br>*CSTIFIGGK*<br>*DGPNALTPPK*<br>*DTSPEQLDEILR*<br>*EAFNNAYLELGGLGER*<br>*EKLLDEEMK*<br>*EVNGDASEAALLK*<br>*FGDRIPADIR*<br>*GFKVDNSSLTGESEPQSR*<br>*IAGLASGLDTGETPIAK*<br>*IATLCNR*<br>*KADIGVAMGISGSDVSK*<br>*KVCEIPFNSTNK*<br>*LIFDNLK*<br>*LIFDNLKK*<br>*LIIVEGCQR*<br>*LLDEEMK*<br>*LLDEEMKEAFNNAYLELGGLGER*<br>*MGAIVAVTGDGVNDSPALK*<br>*MGAIVAVTGDGVNDSPALKK*<br>*MTVAHMWFDNQIIEADTTEDQSGVQYDR*<br>*NLAFFSTNAVEGTAK*<br>*NLEAVETLGSTSTICSDK*<br>*NLEAVETLGSTSTICSDKTGTLTQNR*<br>*NSIFHQGMR*<br>*QAADMILLDDNFASIVTGVEEGR*<br>*SAGIKVIMVTGDHPITAK*	**GDLDDLKQELDIDHHK**<br>**GGQEGVPILK**<br>**GVENTHENPLETK**<br>**GVVIScGDNTVMGR**<br>**ISVEELYQR**<br>**KEVnGDASEAALLK**<br>**LNIPVSEVNPR**<br>**NMVPQFATVVR**<br>**RNPGGWLEQETYY**<br>**SVGIISEGNETVEDIAQRLNIPVSEVNPR**<br>**VLGFcDLMLPSDK**<br>**VLGFcDLMLPSDKFPLGFK**<br>**YQVSIHETEDPNDPR**<br>*AAVPDAVAK*<br>*ADIGVAMGISGSDVSK*<br>*cSTIFIGGK*<br>*DGPNALTPPK*<br>*DTSPEQLDEILR*<br>*EAFNNAYLELGGLGER*<br>*EKLLDEEMK*<br>*EVnGDASEAALLK*<br>*FGDRIPADIR*<br>*GFKVDNSSLTGESEPQSR*<br>*IAGLASGLDTGETPIAK*<br>*IATLcNR*<br>*KADIGVAMGISGSDVSK*<br>*KVcEIPFNSTNK*<br>*LIFDNLK*<br>*LIFDNLKK*<br>*LIIVEGcQR*<br>*LLDEEMKEAFNNAYLELGGLGER*<br>*MGAIVAVTGDGVNDSPALK*<br>*MGAIVAVTGDGVNDSPALKK*<br>*MTVAHMWFDNQIIEADTTEDQSGVQYDR*<br>*NLAFFSTnAVEGTAK*<br>*NLEAVETLGSTSTIcSDK*<br>*nLEAVETLGSTSTIcSDKTGTLTQNR*<br>*NSIFHQGMR*<br>*QAADMILLDDnFASIVTGVEEGR*<br>*RNSIFHQGMR*<br>*SVGIISEGNETVEDIAQR*<br>*VcEIPFNSTNK*

		SVGIISEGNETVEDIAQR<br>*TTPEWVK*<br>*VCEIPFNSTNK*<br>*VDNSSLTGESEPQSR*<br>*VIMVTGDHPITAK*<br>*YHTEIVFAR*	*VDnSSLTGESEPQSR*<br>*VIMVTGDHPITAK*<br>*YHTEIVFAR*
β1			**NQDGYLSPLIAVR**
β2	**AINPELIK**<br>**DFPETmPEDLK**<br>**ENIGEIQYIPR**<br>**EScNLWTK**<br>**EVGFYSAPK**<br>**GQNIYNcDYDRPPGK**<br>**GQVcDVDVK**<br>**KPGLTPGR**<br>**NHILTQK**<br>**STLLWYQGK**<br>**TGVIINVEcR**<br>**TLENFLEVYR**	**AINPELIK**<br>**DFPETMPEDLK**<br>**DFPETMPEDLKNHILTQK**<br>**ENIGEIQYIPR**<br>**ESCNLWTK**<br>**ESLIGQDPGLGFR**<br>**EVGFYSAPK**<br>**GQNIYNCDYDRPPGK**<br>**GQVCDVDVK**<br>**IGIVHFELLID**<br>**KEVGFYSAPK**<br>**KKEVGFYSAPK**<br>**MEANTQEK**<br>**STLLWYQGK**<br>**TGPSWAK**<br>**TGVIINVECR**<br>**TIWVSCEGESPHDR**<br>**TLENFLEVYR**	**AINPELIK**<br>**DFPETMPEDLK**<br>**DFPETMPEDLKNHILTQK**<br>**ENIGEIQYIPR**<br>**EScNLWTK**<br>**EVGFYSAPK**<br>**GQNIYNcDYDRPPGK**<br>**GQNIYNcDYDRPPGKGQVc DVDVK**<br>**GQVcDVDVK**<br>**KEVGFYSAPK**<br>**mEANTQEK**<br>**STLLWYQGK**<br>**TGVIINVEcR**<br>**TIWVScEGESPHDR**<br>**TIWVScEGESPHDRENIGEI qYIPR**<br>**TLENFLEVYR**
**β3**	**DmPEDLK**<br>**DmPEDLKSHINEEK**<br>**EEFYTRPAK**<br>**LFLWNSETSQFLGR**<br>**SWSPcVK**<br>**TFSSWAK**<br>**VGNWDAFK**<br>**VSESNYATWTNK**<br>**YENVmQScDFDTPDTPDR**	**AKEEFYTRPAK**<br>**DMPEDLKSHINEEK**<br>**DMPEDLKSHINEEKK**<br>**EEFYTRPAK**<br>**ENNYAYHNGAPCIFLK**<br>**GFPGFYFPYK**<br>**GSVHFELMID**<br>**LDEFLKPYR**<br>**LDEFLKPYREPDPK**<br>**LFLWNSETSQFLGR**<br>**PMPPESHVESTLIWFK**<br>**RGFPGFYFPYK**<br>**RGSVHFELMID**<br>**SWSPCVK**<br>**TFSSWAK**<br>**VGNWDAFK**<br>**VSESNYATWTNK**<br>**WQQASSLIGSNPGLGFR**<br>**YENVMQSCDFDTPDTPDR**	**AKEEFYTRPAK**<br>**dMPEDLKSHINEEK**<br>**EEFYTRPAK**<br>**ENnYAYHnGAPcIFLK**<br>**GFPGFYFPYK**<br>**LFLWNSETSQFLGR**<br>**LDEFLKPYREPDPK**<br>**SLNMVWVScEGENPADVE NIGSIQYIPR**<br>**SWSPcVK**<br>**VGNWDAFK**<br>**VSESNYATWTNK**<br>**YENVMQScDFDTPDTPDR**

Table S3: Identification of NKA subunits by gel purification and ensuing LC-MS/MS. Counts of unique peptides, sum of unique and shared peptides and sequence coverage identified in each sample of gel 1 and gel 2 for each of the five subunits.

gel	Subunit detected	sample	unique peptides (count)	total peptides (unique and shared) (count)	sequence coverage [%]
gel 1	αA	I	0	22	22.1
		II	1	21	22.2
		III	0	17	20.1
	αB	I	14	43	45.8
		II	11	45	50
		III	7	36	44.4
	αC	I	18	47	49.4
		II	17	47	51.5
		III	14	40	48.9
	β2	i	7	7	18.9
		ii	11	11	33.2
		iii	6	6	16.5
	β3	i	6	6	17.8
		ii	9	9	28.3
		iii	8	8	26.1
gel 2	αA	I	2	33	24.4
		II	4	40	27.7
		III	2	33	24.5
		IV	2	31	23.8
	αB	I	10	67	48.6
		II	10	77	50.2
		III	13	76	52.6
		IV	10	64	48.9
	αC	I	18	75	52.8
		II	20	94	53.8
		III	19	83	55.3
		IV	19	70	53.6
	β2	i	3	3	11.5
		ii	11	11	35.4
		iii	15	15	42.2
		iv	12	12	37
	β3	i	13	13	40.1
		ii	18	18	56.1
		iii	17	17	56.1
		iv	11	11	33.4

Table S4: Results from statistical analyses (ANOVA and TukeyHSD) of differences in amounts of unique peptides, unique PSMs and sequence coverage between the detected subunits (αA, αB, αC, β2 and β3) from the two 2D-gels. Significant results are highligted with bolt letters.

sample	variable	statistical test	results
gel 1	unique peptides	ANOVA	**$F_{4,10}$=19.03, p<0.001**
αA – αB		TukeyHSD	**p=0.002**
αA – αC			**p<0.001**
αB – αC			p=0.076
β2 – β3			p=1
gel 1	unique PSMs	ANOVA	**$F_{4,10}$=10.39, p=0.001**
αA – αB		TukeyHSD	**p=0.016**
αA – αC			**p<0.001**
αB – αC			p=0.302
β2 – β3			p=0.904
gel 1	sequence coverage	ANOVA	**$F_{4,10}$=23.83, p<0.001**
αA – αB		TukeyHSD	**p<0.001**
αA – αC			**p<0.001**
αB – αC			p=0.931
β2 – β3			p=1
gel 2	unique peptides	ANOVA	**$F_{4,15}$=18.25, p<0.001**
αA – αB		TukeyHSD	**p=0.008**
αA – αC			**p<0.001**
αB – αC			**p=0.008**
β2 – β3			p=0.225
gel 2	unique PSMs	ANOVA	**$F_{4,15}$=19.29, p<0.001**
αA – αB		TukeyHSD	**p=0.01**
αA – αC			**p<0.001**
αB – αC			**p=0.004**
β2 – β3			p=0.101
gel 2	sequence coverage	ANOVA	**$F_{4,15}$=9.49, p<0.001**
αA – αB		TukeyHSD	**p=0.004**
αA – αC			**p=0.001**
αB – αC			p=0.961
β2 – β3			p=0.119

Table S5: Similarity and identity between β protein sequences of *O. fasciatus* and *D. melanogaster* expressed in percent [%], calculated by the online tool Sequence Manipulation Suite provided by Stothard (2000). The one that showed highest identity (and similarity) values are highlighted with bold numbers.

Similarity / Identiy [%]	Nrv1	Nrv2	Nrv3
β1	**55.76 / 42.42**	62.57 / 43.41	58. 31 / 40.48
β2	56 /39.38	**72.14 / 58.51**	61.18 / 45.03
β3	55.14 / 39.88	67.8 / 52.32	**70.48 / 59.68**
βx	39.69 / 25.31	45.2 / 32.2	42.36 / 28.03
