# Supplementary material for: Na,K-ATPase α1 and β-subunits show distinct localizations in the nervous tissue of the large milkweed bug
Source: Cell Tissue Res. 2022 Mar 25;388(3):503–19. doi: 10.1007/s00441-022-03580-6 (PMC9110512; doi:10.1007/s00441-022-03580-6)
Supplement: Supplementary file 2 — Supplementary file2 (PDF 8121 KB) [file 441_2022_3580_MOESM2_ESM.pdf]

Supplemental Information

Figures S1-S5

Na,K-ATPase α1 and β-subunits show distinct localizations in the nervous tissue of the large milkweed bug

Cell and tissue research

Marlena Herbertz[1*], Sönke Harder[2], Hartmut Schlüter[2], Christian Lohr[3], Susanne Dobler[1]

[1]Institute of Zoology, Molecular Evolutionary Biology, Universität Hamburg, 20146 Hamburg, Germany

[2]Institute of Clinical Chemistry and Laboratory Medicine, University Medical Center Hamburg-Eppendorf, 20246 Hamburg, Germany

[3]Institute of Zoology, Neurophysiology, Universität Hamburg, 20146 Hamburg, Germany

Corresponding author: Marlena Herbertz

E-mail: marlena-winter@uni-hamburg.de

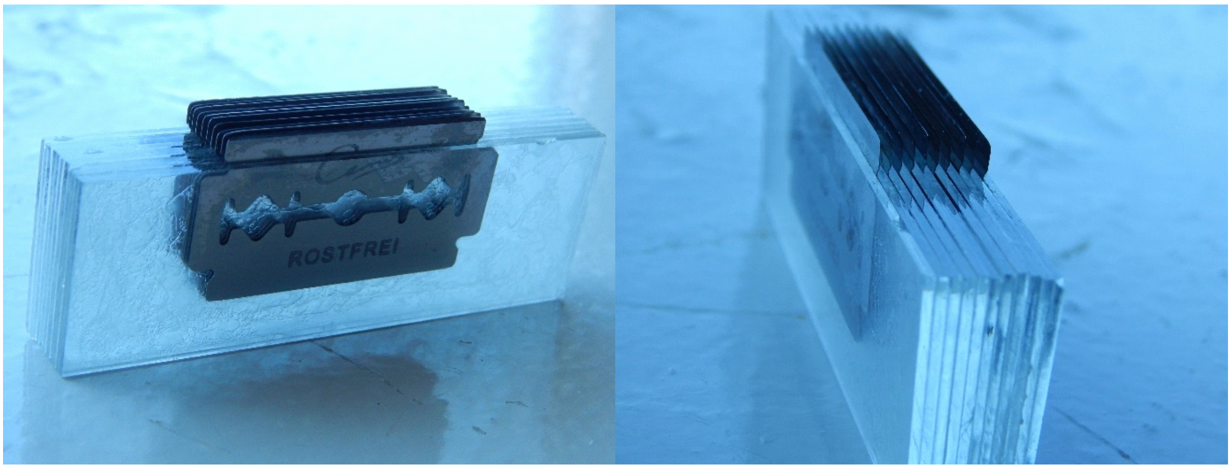

Fig. S1: Home-made cutting tool made of eleven microscope slides and ten razor blades. The razor blades were fixed permanently between the slides to guarantee equal slice width

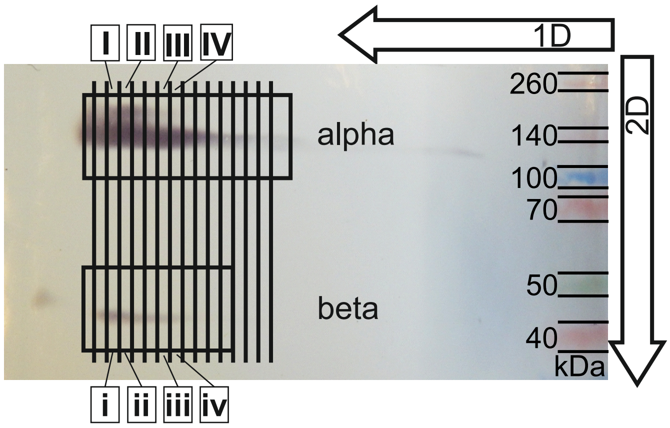

Fig. S2: Western blot of a 2D-(BN/tris tricine) PAGE of nervous tissue protein solution. The staining was performed with α5 and Nrv5F7 primary antibody, detected with HRP-conjugated goat anti-mouse secondary antibody and visualized with 4-CN. The boxes mark the α1 (~110 kDa) and β-bands (~40 kDa) that were cut in the corresponding silver stained gel (not shown here), the lines mark the gel slices and arrows point out to the gel samples, which were sent for LC-MS/MS analyses (α1-subunit samples: I, II, III, IV and corresponding β-subunit samples: i, ii, iii, iv). The large arrows show the running direction of the first and second dimension. (Please note: The nitrocellulose membrane was not exactly straight orientated on the tris tricine gel during the western blotting process and this resulted in slightly lower marks of the BR protein ladder.)

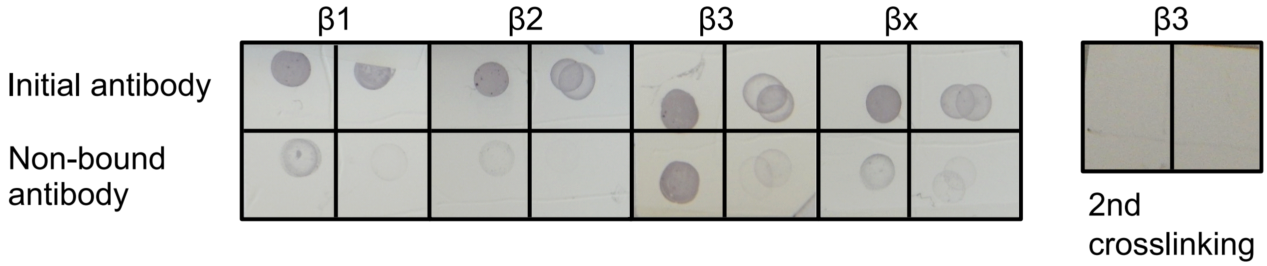

Fig. S3: Evaluation of antibody-magnetic bead cross-linking success via dot blot. Two times 2 µl of undiluted (left column), 1:10 dilution (right column) of initial antibody solution (upper row), and non-bound antibody solution (bottom row) were dripped on a nitrocellulose membrane and detected with HRP-conjugated secondary antibodies (goat anti-rabbit (β2, βx) and goat anti-chicken (β1, β3)) and visualized with 4-CN. All non-bound antibody fractions show a weak signal indicating a successful coupling to the beads except for β3 non-bound antibody solution. After a second crosslinking reaction of the β3 non-bound antibody solution with the magnetic beads no signal on the dot blot was detected (2nd crosslinking, left box undiluted solution, right box 1:10)

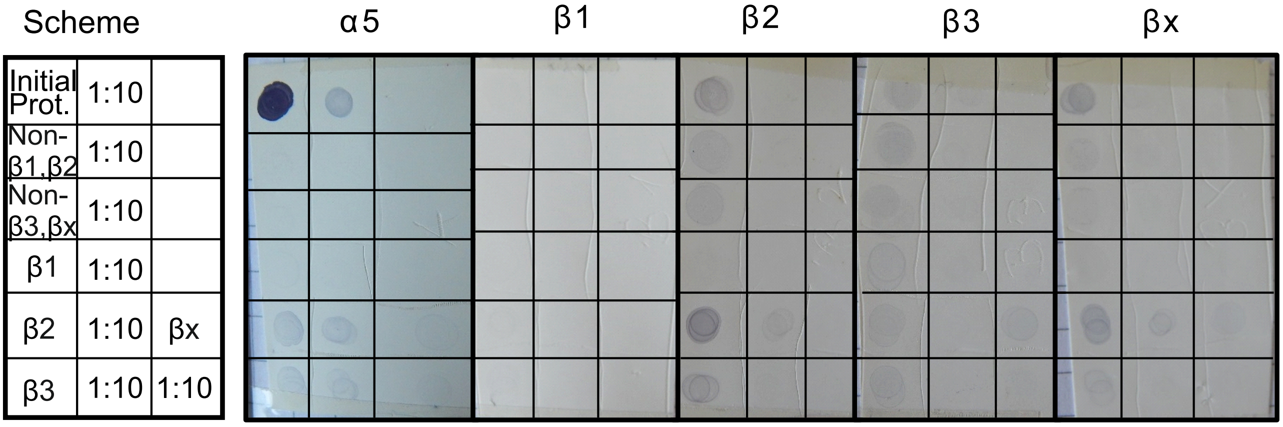

Fig. S4: Verification of target protein presence in the IP eluate via dot blot. 2x 2µl of protein solution (undiluted and 1:10 dilution) were dripped onto nitrocellulose membranes (5 replicates) following the pipetting scheme (left box), stained with alpha specific-α5 and beta-specific β1, β2, β3 and βx antibodies, detected with HRP-conjugated secondary antibodies (goat anti-rabbit (β2, βx) and goat anti-chicken (β1, β3)) and visualized with 4-CN. Besides the four different magnetic bead eluates (β1, β2, β3, βx), the initial protein solution (Initial Prot.) and the protein solution after incubation with β1 and β2 as well as β3 and βx (Non-β1,β2; Non-β3,βx) were tested

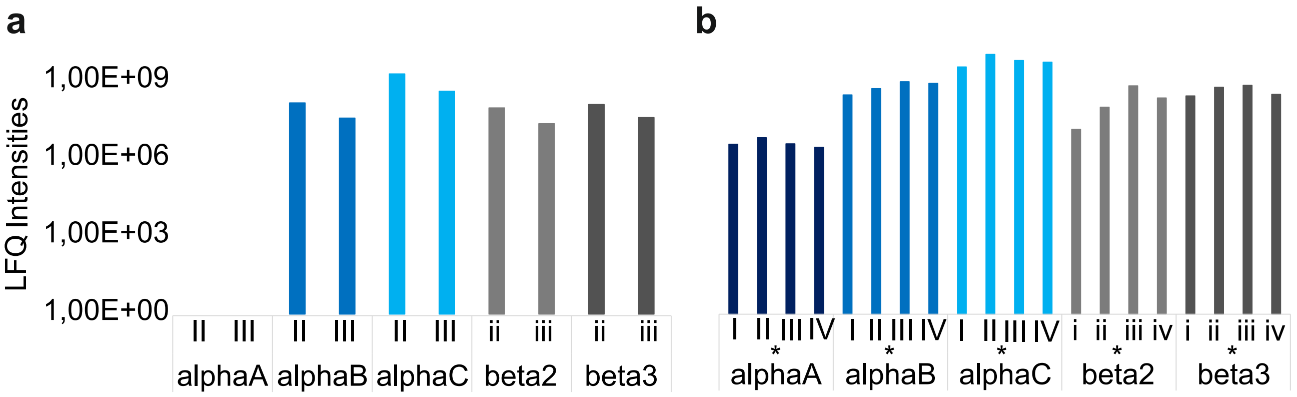

Fig. S5: Lable free quantification (LFQ) intensities of the five identified NKA subunits. a NKA subunits originating from the gel samples I (i), II (ii), and III (iii) of gel 1 (Fig. 1a). **b** NKA subunits originating from the gel samples I (i), II (ii), III (iii), and IV (iv) of gel 2 (Fig. S2). Comparisons of specific subunit intensities can be made across samples but not between different subunits within one sample.
